# Resistance–capacitance optimizer: a physics-inspired population-based algorithm for numerical and industrial engineering computation problems

**DOI:** 10.1038/s41598-023-42969-3

**Published:** 2023-09-23

**Authors:** Sowmya Ravichandran, Premkumar Manoharan, Pradeep Jangir, Shitharth Selvarajan

**Affiliations:** 1 Department of Electrical and Electronics Engineering, Manipal Institute of Technology, Manipal Academy of Higher Education, Manipal, Karnataka 576104 India; 2 Department of Electrical and Electronics Engineering, Dayananda Sagar College of Engineering, Bengaluru, Karnataka 560078 India; 3 Rajasthan Rajya Vidyut Prasaran Nigam Limited, Sikar, Rajasthan 332025 India; 4 Department of Computer Science, Kebri Dehar University, 001 Kebri Dehar, Ethiopia

**Keywords:** Engineering, Mathematics and computing

## Abstract

The primary objective of this study is to delve into the application and validation of the Resistance Capacitance Optimization Algorithm (RCOA)—a new, physics-inspired metaheuristic optimization algorithm. The RCOA, intriguingly inspired by the time response of a resistance–capacitance circuit to a sudden voltage fluctuation, has been earmarked for solving complex numerical and engineering design optimization problems. Uniquely, the RCOA operates without any control/tunable parameters. In the first phase of this study, we evaluated the RCOA's credibility and functionality by deploying it on a set of 23 benchmark test functions. This was followed by thoroughly examining its application in eight distinct constrained engineering design optimization scenarios. This methodical approach was undertaken to dissect and understand the algorithm's exploration and exploitation phases, leveraging standard benchmark functions as the yardstick. The principal findings underline the significant effectiveness of the RCOA, especially when contrasted against various state-of-the-art algorithms in the field. Beyond its apparent superiority, the RCOA was put through rigorous statistical non-parametric testing, further endorsing its reliability as an innovative tool for handling complex engineering design problems. The conclusion of this research underscores the RCOA's strong performance in terms of reliability and precision, particularly in tackling constrained engineering design optimization challenges. This statement, derived from the systematic study, strengthens RCOA's position as a potentially transformative tool in the mathematical optimization landscape. It also paves the way for further exploration and adaptation of physics-inspired algorithms in the broader realm of optimization problems.

## Introduction

### Overview

In computer science, optimization is the procedure of selecting the optimum solutions for a particular system from among all the potential values to optimize the result. As the sophistication of problems has expanded over the last few decades, the demand for innovative optimization approaches has become increasingly critical^1^. In particular, earlier traditional mathematical strategies that have been employed to handle optimization design problems are usually deterministic and suffer from a severe difficulty known as “local optimality trapping.” As a result, these strategies are extremely inefficient when applied to real-world optimization problems, which has increased attention in stochastic optimization algorithms during the last 20 years^2^. Most real-world optimization problems, such as those in engineering, bio-informatics, tuning of machine learning parameters, feature selection, image processing, wireless sensor networks, and other fields, are highly non-linear and non-convex as a result of the presence of complex constraints and a large number of design variables in the problem domain. As a result, solving them is difficult due to many inherent local minima in such optimization problems. Furthermore, there is no certainty that a worldwide solution will be found. In order to overcome the challenges connected with these types of real-world optimization problems, it is necessary to develop new and more effective strategies for finding better solutions. Many researchers have attempted to suggest new algorithms and/or enhance the current ones to arrive at satisfactory results^3^.

As discussed earlier, optimization is a growing research study that deals with maximizing or minimizing the objectives of given optimization problems. Practical optimization problems are often labelled NP-hard issues that traditional computational models cannot adequately or correctly solve^4^. Metaheuristic optimization techniques have become more prominent for solving various problems in different domains to accompany the realities. (i) it depends on basic concepts and is simple to execute; (ii) does not require details on the cost function gradient; (iii) can avoid local optima; and (iv) it is used to handle a multitude of problems in multiple fields. As the implementation of metaheuristic techniques relies on computing facilities, the advancement of metaheuristics has been enhanced by improvements in computational capability^5–8^. The two principal stages of metaheuristics are exploitation and exploration. The significant differences between metaheuristic optimization exist in the primary ways in which those two phases are balanced. Regarding metaheuristic techniques, such as population-based or single-solution-based metaheuristics, the difference around them is a primary concept and is sometimes considered fundamental. Traditional metaheuristics based on single solutions seem to be higher intensification than diversification, whereas metaheuristics based on populations are more diversification than intensification. Due to the stochastic nature of metaheuristic techniques, balancing the two stages is going to be difficult^9,10^.

The introduction of new optimization algorithms is justified by several factors that aim to improve existing methods and address specific challenges. Firstly, new algorithms can enhance efficiency by reducing computational complexity and the time required to find optimal solutions. Techniques like parallel computing, metaheuristics, and machine learning enable algorithms to explore the search space effectively and converge faster. Secondly, complex and large-scale problems often demand more sophisticated approaches. Traditional algorithms may struggle due to computational limitations, making it necessary to develop new algorithms, such as evolutionary algorithms and swarm intelligence, to handle these challenges. Thirdly, robustness and flexibility are crucial in optimization. New algorithms incorporate techniques to handle uncertainties, noisy data, and dynamic environments, ensuring more reliable and adaptable solutions^11,12^.

Additionally, many optimization problems have multiple local optima that can trap traditional algorithms. Novel algorithms introduce exploration and exploitation techniques to overcome local optima and improve the chances of finding the global optimum. Furthermore, problem-specific optimization algorithms are developed to exploit domain-specific knowledge and problem structures. These algorithms offer significant improvements by leveraging insights from specific domains like scheduling, network optimization, or supply chain management. Lastly, advancements in computing power enable new algorithms to handle larger problem instances and perform more extensive searches. Algorithms can utilize parallel, distributed, or cloud computing to expedite the optimization process. Overall, the justifications for introducing new optimization algorithms include improved efficiency, handling complex problems, robustness, overcoming local optima, problem-specific optimization, and leveraging advancements in computing power. These justifications drive progress in optimization research, providing more effective solutions to real-world problems^13–15^.

### Literature study

An established subgroup of the evolutionary algorithm consists of optimization methods inspired by nature that have immense potential to address such combinatorial optimization challenges. The domain of nature-inspired optimization algorithms has seen many studies in the earlier few decades. The investigators discussed developments throughout every area of life and Darwinian selection optimization approaches. Human-based algorithms (HBA), physics-based algorithms (PBA), swarm-based algorithms (SBA), and evolutionary algorithms (EA) are the four main categories under which nature-inspired optimization algorithms are categorized. A few advanced and famous techniques from each branch are discussed as follows. The natural law processes inspire the EA. Initialization is the first step in EA in a random search location, and then successive iterations keep evolving. These techniques determine the optimal population in a generation and merge to create the next population generation. Thus, the solution accuracy of the population is enhanced over iterations. The most prominent EA approach is the genetic algorithm (GA), replicating Darwin’s theory^16^. Each solution is portrayed as a chromosome in a GA, and the chromosomes with a maximum fitness value are often traversed with other chromosomes in every generation. Therefore, over generations, the overall fitness of all chromosomes improves. Evolution Strategy (ES)^17^, Biogeography-Based Optimizer (BBO)^18^, and Differential Evolutionary (DE)^19^ are among the algorithms in this group.

SBAs are frequently motivated by the coordinated intelligent actions of living creatures. Living things interrelate with each other in everyday life to attain the highest possible combined efforts. In this category, Particle Swarm Optimization (PSO) is among the utmost effective methods^20^. The PSO algorithm is driven by the food-searching actions of the birds' swarms^21^. Another important technique in this group is the Ant Colony Optimization (ACO) algorithm, which is motivated by the direction of the food source of ants seeking behaviour from the colony^22^. Krill Herd (KH)^23^, Cuckoo Search Algorithm (CSA)^24^, Ant Lion Optimizer (ALO)^25^, Artificial Bee Colony (ABC)^26,27^, Bat Algorithm (BA)^28^, Firefly Algorithm (FA)^29,30^, Grey-Wolf Optimizer (GWO)^31,32^, Manta-Ray Foraging Optimization (MRFO)^33^, Salp Swarm Optimization (SSA)^34^, artificial rabbit optimizer^35^, Whale Optimization Algorithm (WOA)^36^, moth flame optimization^37^, and Marine-Predator Algorithm (MPA)^38^ are few examples of swarm-based algorithms that are recently reported.

The third cluster, known as PBA, consists of computations that apply physical principles. For instance, the Big-Bang Crunch (BBC) is based on the two leading theories of the universe's evolution^39^. The Simulated Annealing (SA) is motivated by metal hardening, including controlled cooling of a substance and heating to expand the crystals' size and decrease the defect density^40^. The Gravitational Search (GS) method is motivated by the law of interactions of mass and gravity^41^, the Small-World Optimization (SWO) algorithm^42^, the Galaxy-Based Search (GBS) algorithm^43^, Quantum-inspired Genetic (QG) algorithm^44^, Sine–Cosine Algorithm (SCA)^45^, atom search algorithm^46^, Crystal Structure Algorithm (CrSA)^47^, Hysteretic Optimization (HO) algorithm^48^, Central Force Optimization (CFO) algorithm^49^, Charged System Search (CSS) algorithm^50^, and Gravitational Local Search (GLS) algorithm^51^ are other well-known algorithms.

**Figure 1:**
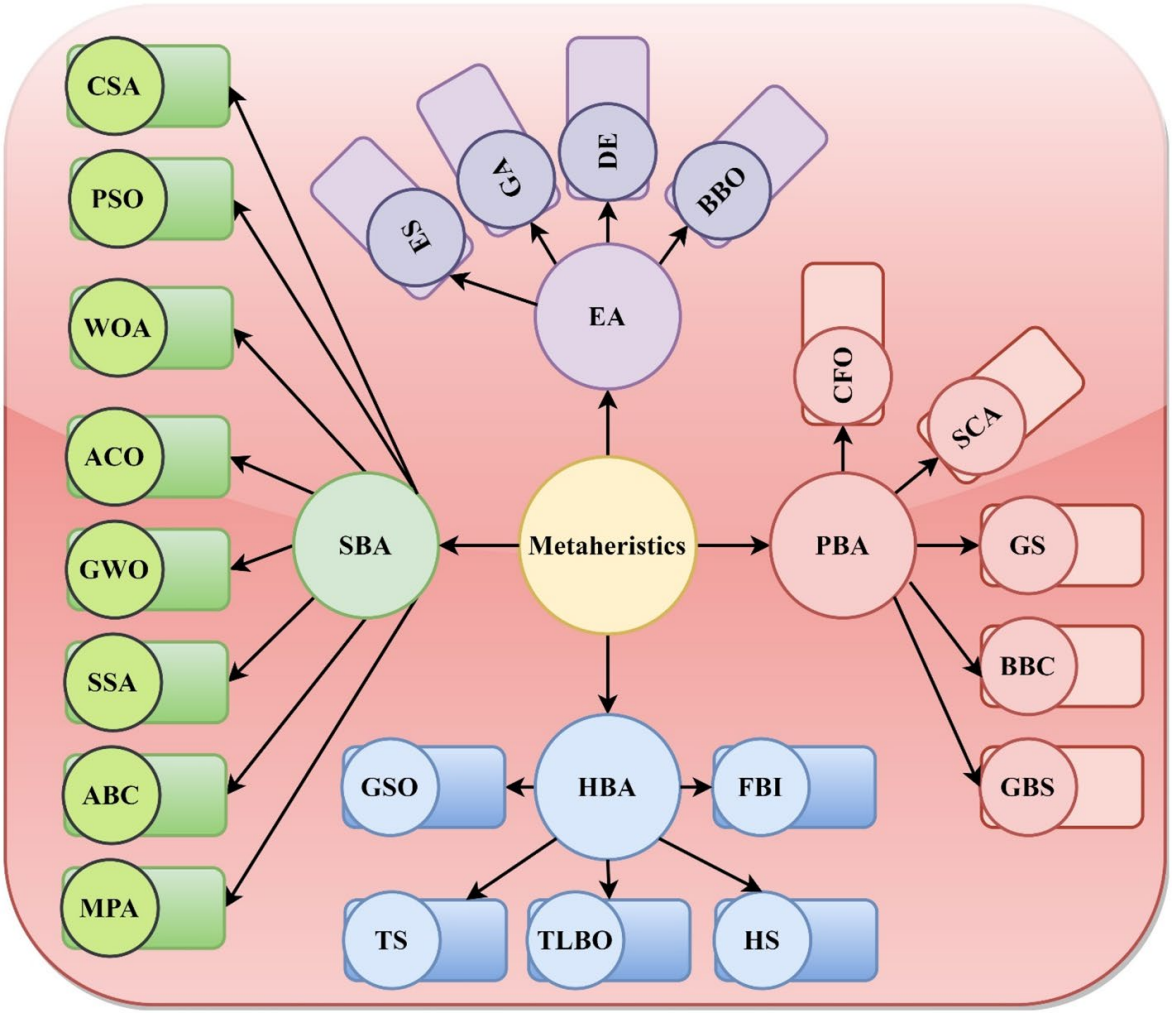
A brief classification of metaheuristic algorithms.

In addition to methods motivated by nature, another class of metaheuristic algorithms known as human activities influenced HBA. Harmony Search (HS) is an analogy to the musical method of looking for a balanced state of equilibrium to find solutions to optimization problems^52^. The Teaching–Learning-Based Optimization (TLBO) algorithm is perhaps the most common technique in this category based on activities necessary in teaching–learning, wherein the teacher impacts the students' performance in the classroom. Both teaching and self-learning impact the outcome of the students^53^. Forensic-Based Investigation (FBI) algorithm is influenced by police officers involved in forensic investigations in the suspicious activity location-pursuit method^54^. Election-Campaign (EC) algorithm^55^, Tabu Search (TS) algorithm^56^, Artificial Immune Algorithm^57^, Social-Based Algorithm (SBA)^58^, League Championship (LC) algorithm^59^, Political Optimizer (PO)^60^, Group Search Optimization (GSO) algorithm^61^, RAO^62^, and Imperialist Competitive (IC) algorithm^63^ are other standard optimizations. The pictorial illustration of metaheuristic classification is shown in Fig. 1.

In contrast to all the aforementioned advanced methods, many population-based algorithms have been developed recently. The following are a few common swarm-based metaheuristic strategies that have recently been developed. The Squirrel Search (SS) algorithm is stimulated by the searching actions of flying squirrels^64^, the Seagull Optimization (SO) algorithm is stimulated by the seagull’s movement and attacking actions^65^, the Tunicate Swarm Algorithm (TSA) is stimulated by the swarming behaviour of tunicates at sea^66^, Emperor Penguins Colony (EPC) algorithm stimulated by the movement of penguins^67^, Colony Predation Algorithm (CPA) stimulated by corporate predation of animals^68^, Butterfly Optimization (BO) algorithm (BOA) stimulated by butterflies food searching and matting actions^69^, and Harris Hawk Optimization (HHO) algorithm stimulated by supportive and chasing actions of Harris’ hawks^70^. Furthermore, additional human-based metaheuristic techniques are named as follows. Poor and Rich Optimization (PRO) algorithm^71^, Team Game (TG) algorithm stimulated by game plans used in volleyball, basketball, and football^72^, and Deer Hunting Optimization (DHO) algorithm stimulated by human beings’ hunting approaches^73^. The mathematical operators inspired the Arithmetic Optimization Algorithm (AOA)^74^. Besides, the latest progress in the area of optimization techniques involves, but is not restricted to, the PO method stimulated by the political system^75^, the Equilibrium Optimizer (EO) algorithm stimulated by mass balance models of control volume being used in estimating the both dynamic and equilibrium conditions^76^, Henry Gas Solubility Optimization (HGSO) algorithm stimulated by the law of Henry’s^77^, Slime Mould Optimization (SMO) algorithm stimulated by the slime mould’s oscillation mode^78^, and Artificial Electric Field (AEF) algorithm stimulated by Newtons’ law of motion and Coulombs’ law of electrostatic force^79^.

Mostly, metaheuristic algorithms are used to solve real-world engineering problems. For instance, non-linear MPA is used for Hammerstein autoregressive exogenous systems^80^, chaotic GWO is used in identification of control autoregressive model^81^, MPA with key term separation technique used for nonlinear Hammerstein system identification^82^, Dwarf mongoose algorithm for identification of autoregressive exogenous model^83^, Aquila optimizer for control autoregressive systems identification^84^, etc. Though the algorithms are used for real-world applications, still most of the algorithms struggle to handle constraint optimization problems.

### Motivation

All the mentioned metaheuristic algorithms are tested using optimization problems and different engineering optimization problems. The inventors of the algorithms mentioned above have claimed that their algorithms best handle specific problems. However, anyone algorithm cannot solve all types of optimization problems. This was also stated and proved in No-Free-Lunch (NFL) theorem^85^. As per the NFL theory, no single algorithm can solve all optimization problems.

The Resistance Capacitance Optimization Algorithm (RCOA) proposal is primarily motivated by the need for more efficient and reliable optimization algorithms, particularly for numerical and engineering design optimization problems. Traditional algorithms often face limitations such as premature convergence to local optima, inability to handle multimodal and complex optimization landscapes and requirement of various tuning parameters. By mimicking the time response of resistance–capacitance circuits, the RCOA overcomes these challenges with its unique exploration and exploitation phases, absence of control parameters, and ability to handle complex multimodal functions. Physically-inspired or physics-based optimization techniques, like the RCOA, draw motivation from natural phenomena and processes. They are often lauded for their simplicity, adaptability, robustness, and ability to solve a diverse range of complex optimization problems. Several noteworthy examples, such as SA, PSO, GS algorithm, etc., demonstrate the wide applications, such as operational research, computer science, artificial intelligence, neural network training, fuzzy system control, robotics, feature selection, tuning of fuzzy systems, image processing, structural design optimization, water network design, routing, scheduling problems, etc., of these techniques. These diverse applications underscore the versatility and robustness of physics-based optimization techniques.

Regarding RCOA, the goal is to build upon these successes while addressing the limitations often associated with metaheuristics. Given its initial promising results, the RCOA represents a significant step forward in evolving physics-inspired optimization techniques. In addition, most of the above techniques are motivated by nature and humans, and only very limited physics-based algorithms are proposed. This motivated us to propose a new PBA to solve engineering optimization problems based on the transient and steady-state responses of the series connected resistance–capacitance circuit named the Resistance–Capacitance Optimization Algorithm (RCOA). The reported RCOA is not tested on global optimization and constrained engineering design problems. It motivated us to investigate the performance of the RCOA for constrained problems. In addition, the balance between exploration and exploitation is also analyzed using standard unimodal and multimodal benchmark test functions.

### Major contributions

Furthermore, the main contribution of this study is as follows.

- The proposed RCOA is thoroughly evaluated on 23 standard benchmark problems, including unimodal, multimodal, and fixed and variable dimensions features. This paper included detailed performance metrics in the manuscript to illustrate the specific improvements achieved by RCOA explicitly.
- A penalty function based constraint handling mechanism is employed in this study. This feature uniquely equips the RCOA to handle constrained optimization problems, broadening its applicability and effectiveness.
- The RCOA is applied to eight real-world constraint engineering design optimization problems to demonstrate its practical utility. These tests highlight RCOA's versatility and provide insights into its potential applications in the industry.
- This study also establishes the superior performance of the RCOA through direct comparison with seven established optimization techniques. The specific areas of superiority and the corresponding quantifiable gains achieved by RCOA are clearly defined in this study.
- To further corroborate the effectiveness of RCOA, this study conducted Friedman’s rank test and Wilcoxon's rank sum test. These rigorous statistical methods offer a robust confirmation of RCOA's superior performance, thereby enhancing the credibility of our findings.

### Organization of the paper

The paper is organized as follows. In Section “Modelling of the resistance–capacitance optimization algorithm”, the suggested RCOA's inspiration and modelling are discussed, and in Section “Resistance capacitance optimization algorithm (RCOA)”, the suggested RCOA's effective implementation is described. Section “Simulation results and discussions” deeply investigates the performance of the RCOA using the experimental results obtained for 23 benchmark functions. Section “Real-world engineering design optimization problems” discusses the penalty-based constrained handling mechanism and the application of the RCOA in solving constraint engineering design optimization problems. Lastly, Section “Conclusions and future scopes” concludes the paper.

## Modelling of the resistance–capacitance optimization algorithm

This section comprehensively discusses the mathematical modelling of the Resistance–Capacitance Optimization Algorithm (RCOA).

### Kirchhoff’s law of current (KCL)

**Figure 2:**
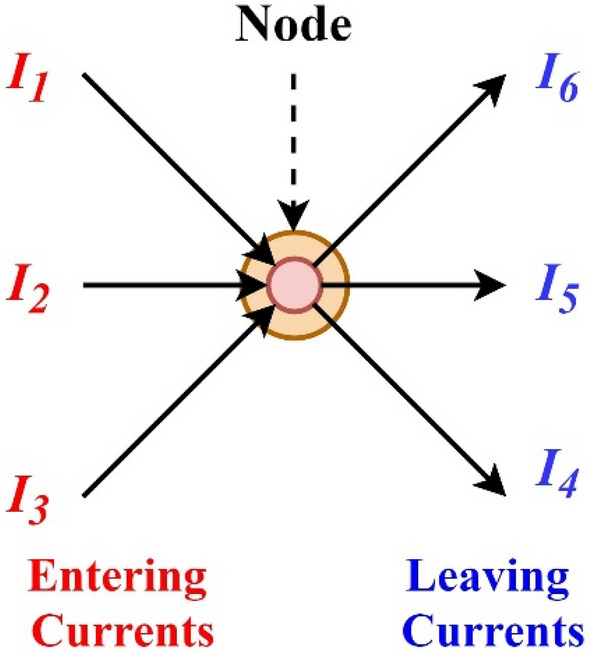
Illustration of the KCL.

KCL is commonly known as the Conservation of Charge, as the current is conserved around the junction with no current loss. In other words, “There is exactly the amount of current or charge approaching a junction or node as there is exiting it as it has no other place to go except to leave, as no charge is lost within the node^86^”. It is mentioned that all currents entering and exiting the junction must add up to zero algebraically as shown in Fig. 2.

In Fig. 2, the three currents, such as *I*_*1*_, *I*_*2*_, and *I*_*3,*_ are entering the nodes, and their magnitude is positive. The three currents, such as *I*_*4*_, *I*_*5*_, and *I*_*6,*_ leave the nodes, and their magnitude is negative. From Fig. 1, the total current equation is expressed as follows.

1 $${I}_{1}+{I}_{2}+{I}_{3}-{I}_{4}-{I}_{5}-{I}_{6}=0.$$

The term Node in Fig. 2 refers to a junction or connection of two or more current elements or paths, such as components and cables.

### Mathematical concepts

**Figure 3:**
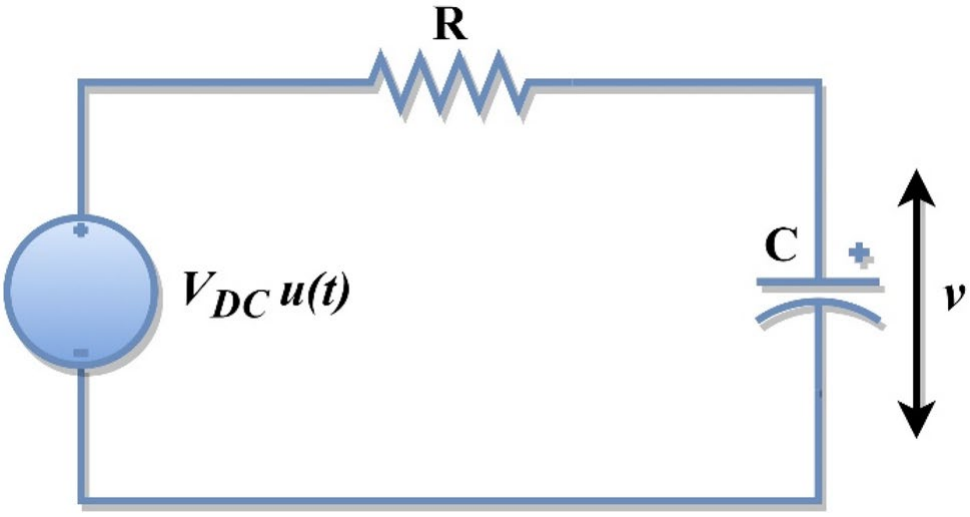
Resistance–capacitance circuit with DC input voltage.

The modern physics approach to the transient behaviour of the resistance–capacitance circuit is given in many textbooks, e.g., Refs.^87–89^. The current or voltage source is modelled as a step function when the direct-current (DC) source is instantly applied to the resistance–capacitance (RC) circuit, and the state is described as a step response. The step response is caused by the sudden application of a DC voltage to the resistance–capacitance circuit, which is depicted in Fig. 3. The initial voltage across the capacitor is denoted as ${v}_{0}$. In the meantime, the voltage across the capacitor cannot change suddenly,

2 $$v\left({0}^{-}\right)=v\left({0}^{+}\right)={v}_{0},$$

where $v\left({0}^{-}\right)$ signifies the capacitor voltage before closing the switch and $v\left({0}^{+}\right)$ signifies the capacitor voltage after the voltage is applied by closing the switch. Here, the voltage across the capacitor is selected as the response of the circuit response to be calculated. The initial voltage across the capacitor is considered even though it is not required to analyze the RC circuit's step response. Apply Kirchhoff’s current law to Fig. 3, and the current equation is expressed as follows.

3 $$\left.\begin{array}{c}\begin{array}{c}C\frac{dv}{dt}+\frac{v-{V}_{s}u\left(t\right)}{R}=0\\ (\mathrm{or})\end{array}\\ \frac{dv}{dt}+\frac{v}{RC}=\frac{{V}_{s}}{RC}u\left(t\right)\end{array}\right\}.$$

The capacitor voltage when the time *t* is greater than zero is denoted as *v*. For $t>0$, Eq. (3) is modified and presented in Eq. (4).

4 $$\frac{dv}{dt}+\frac{v}{RC}=\frac{{V}_{s}}{RC}.$$

Rearrange Eq. (4), and the equation is simplified as follows.

5 $$\frac{dv}{(v-{V}_{s})}=-\frac{dt}{RC}.$$

Integrate both sides of Eq. (5) and introduce the initial condition in Eq. (5), and the final equation is written as follows.

6 $$\mathrm{ln}\frac{v-{V}_{s}}{({v}_{0}-{V}_{s})}=-\frac{t}{RC}.$$

Taking exponential on both sides of Eq. (6), the voltage across the capacitor is written as follows.

7 $$v\left(t\right)={V}_{s}+\left({v}_{0}-{V}_{s}\right){e}^{-\frac{t}{\tau }},$$

where the initial voltage across the capacitor is denoted as ${v}_{0}$ at *t* = 0, $\tau$ is a time constant of the RC circuit and is equal to *RC* and $v\left(\infty \right)$ is the steady-state voltage when *t* > 0. This is referred to as the RC circuit's complete response to a sudden DC voltage application, assuming that the capacitor is primarily charged. Thus, the complete response of the RC circuit is expressed as follows^87–89^.

8 $$v\left( t \right) = \left\{ {\begin{array}{ll} {v_{0} ,} & {t < 0} \\ {V_{s} + \left( {v_{0} - V_{s} } \right)e^{{ - \frac{t}{\tau }}} ,} & {t > 0} \\ \end{array} } \right..$$

**Figure 4:**
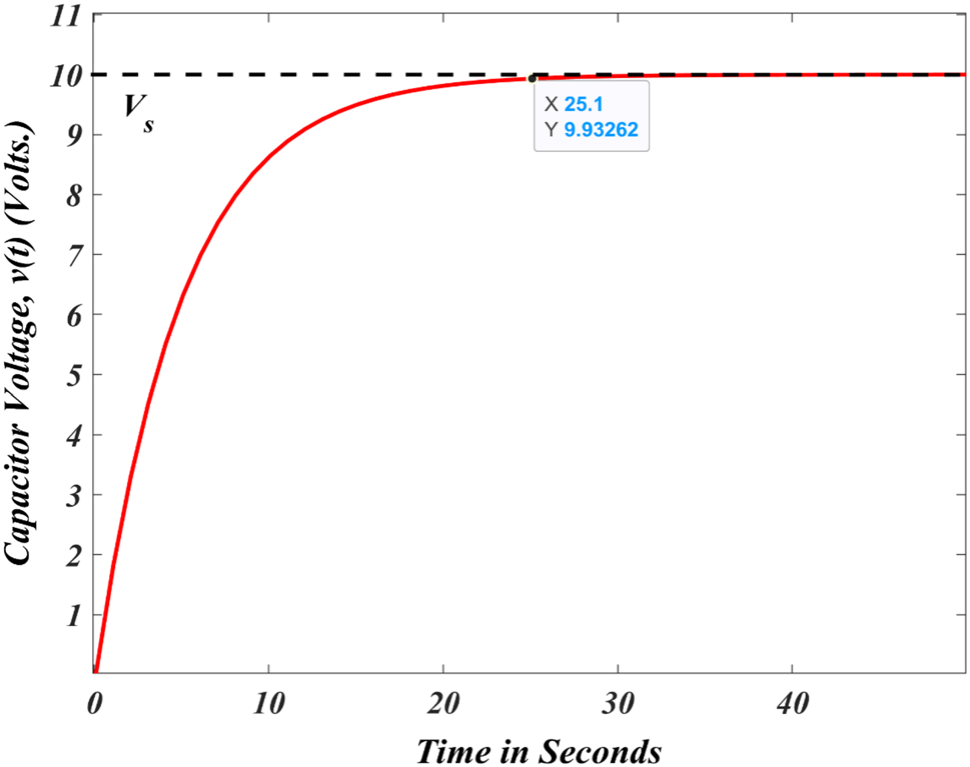
Voltage across the capacitor with zero initial voltage.

The complete resistance–capacitance circuit response is illustrated in Fig. 4 by considering the value of $R=2.5 k\Omega$, $C=1mF$, and $Vs=10 V$ (step signal) by assuming ${V}_{s}<{v}_{0}$. Consider the initial voltage across the capacitor is zero, i.e., ${v}_{0}=0 V$. Equation (8) is modified by substituting ${v}_{0}=0$ and written in Eq. (9)^87–89^.

9 $$v\left( t \right) = \left\{ {\begin{array}{ll} {0,} & {t < 0} \\ {v_{i}^{{Final}} \times \left( {1 - e^{{ - \frac{t}{\tau }}} } \right),} & {t > 0} \\ \end{array} } \right..$$

Equation (9) can be rewritten as presented in Eq. (10).

10 $$v\left(t\right)={V}_{s}\times \left(1-{e}^{-\frac{t}{\tau }}\right)\times u\left(t\right).$$

**Figure 5:**
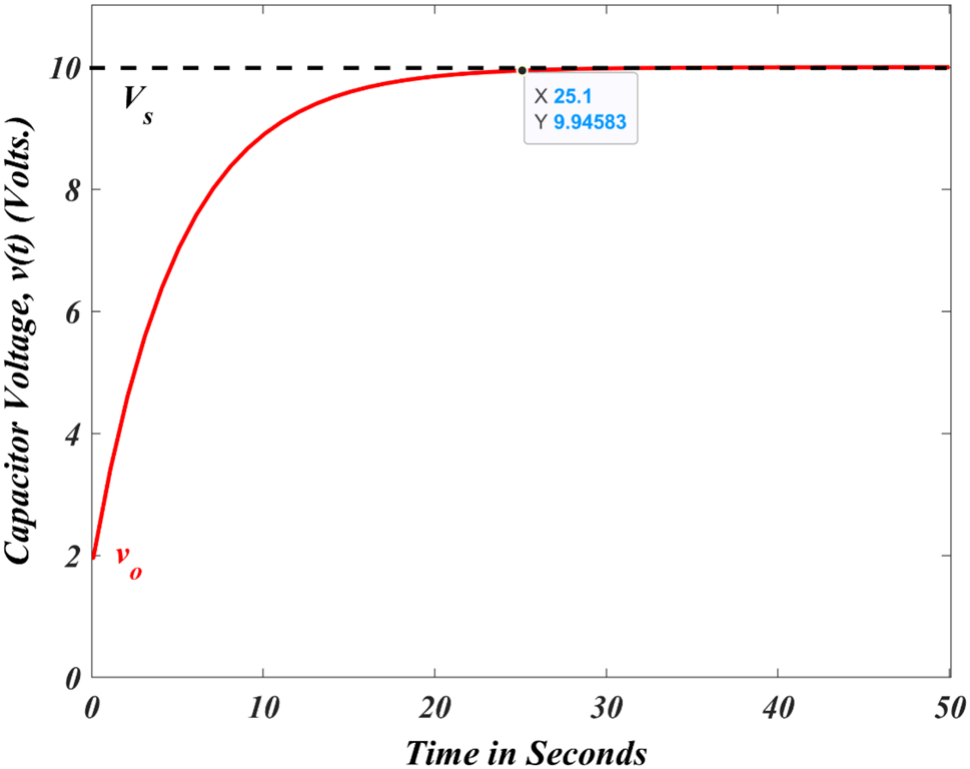
Voltage across the capacitor with an initial voltage.

Equation (10) considers the starting capacitor voltage to be zero and provides the entire response of the resistance–capacitance circuit. In Fig. 5, the resistance–capacitance circuit response is shown with an initial capacitor voltage (${v}_{0}=2 V$).

More accurately, there is a comprehensive procedure for determining the dynamic response of an RC circuit rather than going through the previous equations. Let's take another look at Eq. (7), which is a little more comprehensive than Eq. (10). It is clear that there are two halves to it. There are two methods to break this down into two portions in the traditional sense. The first is to divide it into transient and steady-state responses or forced and natural responses and Eqs. (11), (12) express the total response of the RC circuit.

11 $$\mathrm{Complete\, Response}=\mathrm{Natural\, Response}+\mathrm{Forced\, Response},$$

or

12 $$\left.\begin{array}{l}v={v}_{natural}+{v}_{forced}\\ {v}_{natural}={v}_{o}\times {e}^{-\frac{t}{\tau }}\\ {v}_{forced}={V}_{s}\times \left(1-{e}^{-\frac{t}{\tau }}\right)\end{array}\right\}.$$

Because of the exponential decay of the initial voltage, the output of the RC circuit is a function of time. Because the response is caused by the initial energy stored in the circuit and the physical aspects of the circuit rather than by an external source, it is referred to as the natural response ${v}_{natural}$. ${v}_{forced}$ is referred to as the forced response since it is generated by the circuit when an external source is applied. It depicts what the circuit is compelled to do due to the input stimulation. Eventually, the transient component disappears, keeping only the steady-state component.

Similarly, another way to look at the entire response is to split it into two phases: transient (or temporary) and steady-state (or permanent), as shown in Eqs. (13), (14).

13 $$\mathrm{Complete\, Response}=\mathrm{Transient \,State\, Response}+\mathrm{Steady \,State \,Response},$$

or

14 $$\left.\begin{array}{l}v={v}_{ts}+{v}_{ss}\\ {v}_{ts}={v}_{o}\times {e}^{-\frac{t}{\tau }}\\ {v}_{ss}={V}_{s}\times \left(1-{e}^{-\frac{t}{\tau }}\right)\end{array}\right\}.$$

Because it is fleeting, the transient response ${v}_{ts}$ is defined as the portion of the overall behaviour that decays to zero as the duration reaches infinity. The portion of the total response that persists after the transitory response has died out is referred to as the steady-state response ${v}_{ss}$.

The origin of the responses is the first decomposition of the complete response, whereas the permanence of the responses is the second decomposition. The natural and transient responses are similar in some situations. The steady-state and forced responses are similarly affected. In any case, the whole solution in Eq. (7) could be stated as follows.

15 $$v\left(t\right)=v\left(\infty \right)+\left({v}_{0}-v\left(\infty \right)\right){e}^{-\frac{t}{\tau }}, t>0,$$

or

16 $${v}_{i}^{new}={v}_{i}^{Final}+\left({v}_{i}^{Initial}-{v}_{i}^{Final}\right){e}^{-\frac{t}{\tau }}, t>0,$$

where ${v}_{0}$ or ${v}_{i}^{Initial}$ denotes the initial voltage at $t={0}^{+}$ and $v\left(\infty \right)$ or ${v}_{i}^{Final}$ denotes the steady-state value. Therefore, finding the overall response necessitates the following.

- The initial voltage across the capacitor ${v}_{0}$
- The final capacitor voltage $v\left(\infty \right)$
- The time constant $\tau =RC$.

Item 1 is obtained from the circuit for $t<0,$ and items 2–3 from the circuit for $t>0$. Following determining these elements, we use Eqs. (15) or (16) to calculate the response^90,91^.

### RC circuit charging characteristic

**Figure 6:**
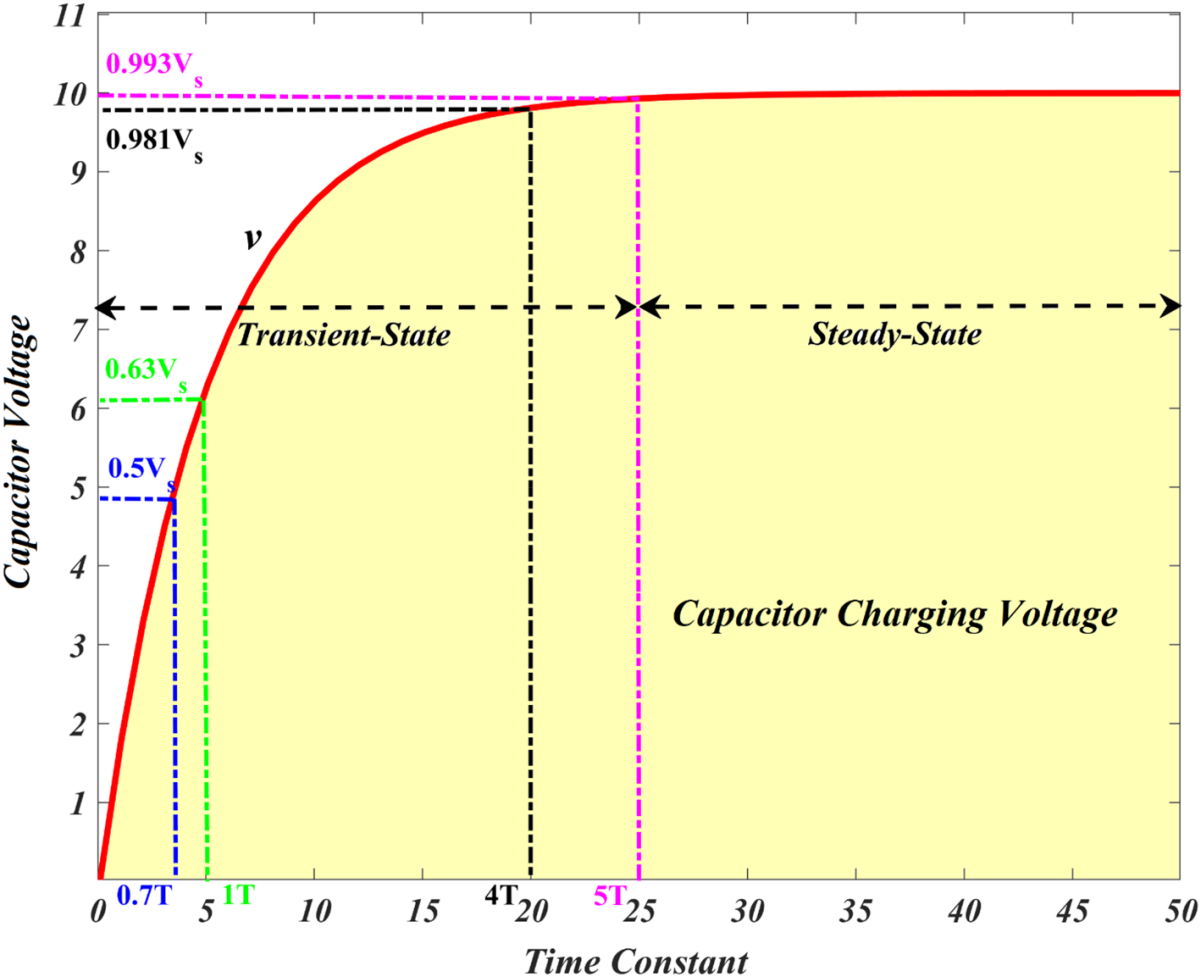
Capacitor charging response.

According to Fig. 6, the capacitor charges at the rate indicated. Since the charging rate is maximum, the charging response of the RC circuit increases more sharply at the start of the charge, but this rises exponentially as the capacitor considers an extra charge at a moderate speed as the charge accumulates^87^.

During the charging, the potential difference between the capacitor's plates increases in proportion to the amount of time it considers to charge up to 63 percent of its allowable voltage ($0.63{V}_{s}$), identified as the one-complete time constant ($T$). The notation 1* T* refers to the $0.63{V}_{s}$ voltage step (one-time constant). As the voltage differential between ${V}_{s}$ and $v$ reduces the capacitor charges. When the capacitor reaches its optimum state, which is higher than five times constants ($5T$), it is considered to be fully charged, $t=\infty$ and $q=Q=CV$. The charging current gradually decreases to zero after approaching infinite, and the capacitor then operates as an open circuit with the input source ${v=V}_{s}$ supplied across it. Furthermore, the mathematical expression for the duration needed for a capacitor charging to $1T$, is as follows:

17 $$\mathrm{RC time constant}, \tau =RC.$$

The time constant only denotes the charge rate where R and C are defined in Ω and Farads, respectively. When the capacitor has been charged for four-time constants ($4T$), the potential difference between the capacitor plates has attained 98% of the input voltage (0.98 ${V}_{s}$), it is reported to be almost fully charged. The transient duration is the time it takes for the capacitor to achieve the $4T$ level after being charged. After $5T$, the capacitor has been charged completely equal to the ${V}_{s}$. Since the capacitor is charged completely, no additional charging current goes through the circuit. Table 1 displays the capacitor voltage of the RC circuit for a specific time constant.

**Table 1:** The maximum output voltage across the capacitor for a different time-constant.

RC value	Time constant	Maximum voltage in %
0.5RC	0.5* T*	39.3
0.7RC	0.7* T*	50.3
1RC	1* T*	63.2
2RC	2* T*	86.5
3RC	3* T*	95.0
4RC	4* T*	98.2
5RC	5* T*	99.3

It is important to note that the charging trajectory for the circuit is exponential rather than linear in nature. In practice, this means that the capacitor is never completely charged to its maximum capacity. Practically, it attains 99.3% after $5T$, at which point the capacitor is deemed to be completely charged. It is possible to compute *v* at any instant because the value of *v* varies with time and has a slight difference at every time constant till 5* T*.

## Resistance capacitance optimization algorithm (RCOA)

This segment explains how the suggested algorithm is developed using the RC circuit response characteristics.

### Implementation of RCOA

As discussed earlier, the total response is decomposed into phases. One is a transient or temporary phase, and the other one is a steady-state or permanent phase. There are two important phases for an optimization algorithm, called exploration and exploitation phases. During the exploration stage, the algorithm looks for the best solution in the search location. Once the algorithm finds the best solution, the algorithm tries to reach the optimal solution in the exploitation phase. Therefore, in the RCOA, the transient phase is mapped with the exploration phase, and the steady-state phase is mapped with the exploitation phase^92^. For a better algorithm, these two phases should balance properly. As mentioned earlier, the response of the RC circuit has a component called ${v}_{o}\times {e}^{-\frac{t}{\tau }}$ (exploration or transient phase), and the response of the RC circuit has a component called ${V}_{s}\times \left(1-{e}^{-\frac{t}{\tau }}\right)$ (exploitation or steady-state phase). The time *t* controls the balancing of these two phases, and the expression for the time *t* is given in Eq. (18).

18 $$t=\left(\frac{it}{Maxit}\right),$$

where $it$ denotes the current iteration, and $Maxit$ denotes the maximum number of iterations. The time *t* linearly increases from 0 to 1.

**Figure 7:**
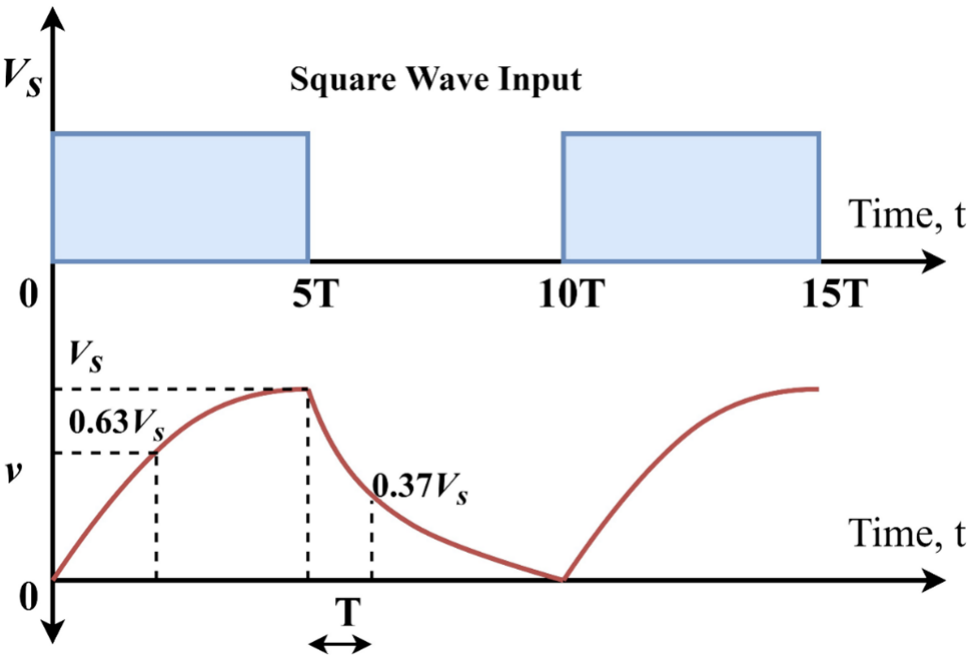
Discharging and Charging profile (5RC time constant).

The charging and discharging of the capacitor in the RC circuit takes a 5* T* period when the source is connected or removed. Assume that square wave input is given to the RC circuit. The RC waveform (capacitor charging and discharging) is shown in Fig. 7.

When the input voltage is increased to a certain level, the voltage across the capacitor swings between + *V*_*s*_ and 0, in this particular case, the frequency of the input voltage waveform is approximately twice that of the 5RC time constant, which is a good fit.

**Figure 8:**
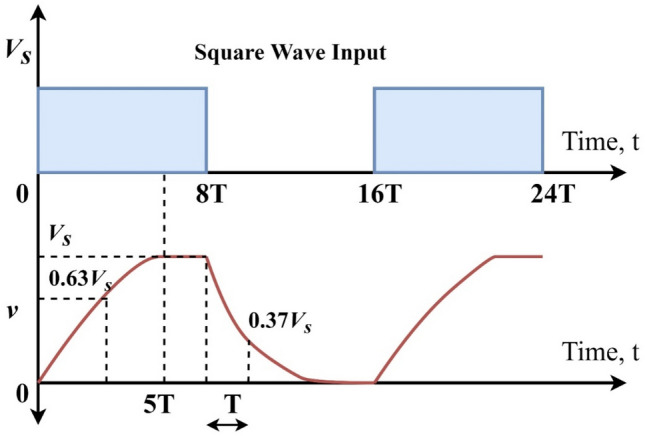
Discharging and Charging profile (8RC time constant).

If the time of the input signal is extended, for instance, by changing the time constant to 8RC, the capacitor would then remain fully charged for a longer period and remain fully discharged for a longer period, producing the waveform as illustrated in Fig. 8.

**Figure 9:**
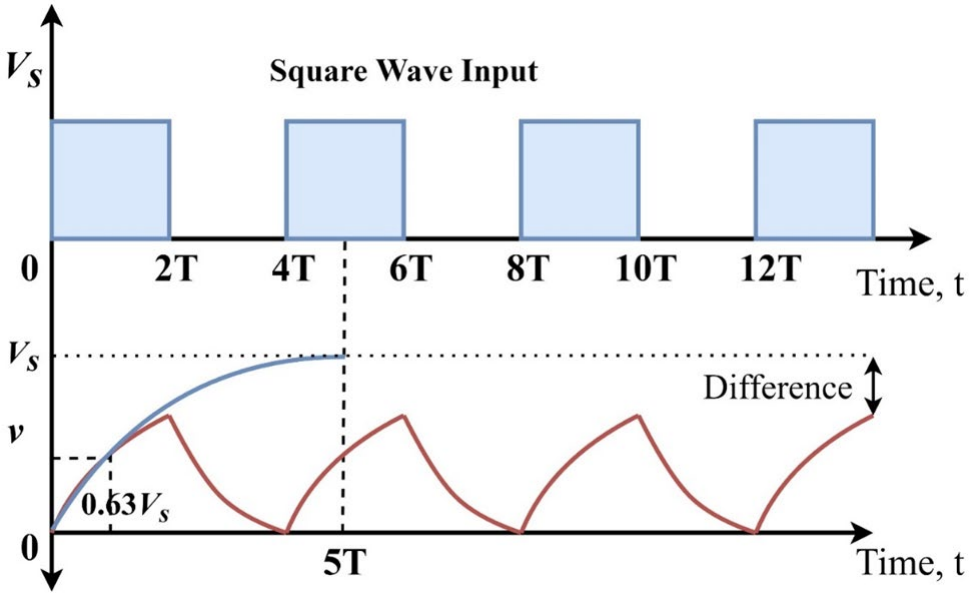
Charging and discharging profile (4RC time constant) of the capacitor.

If lower the overall period of the input waveform is, for example, by changing the time constant to 4RC, the capacitor would not have enough time either to fully charge during the ON-period or fully discharge during the OFF-period, as shown in Fig. 9. To produce an RC waveform, the resultant voltage drop across the capacitor must be smaller than the maximum input voltage of the capacitor.

From the above discussions, the capacitor voltage can then be varied by adjusting the time constant or the input frequency, resulting in a relationship between *v* and *t*. This statement is very important to develop the RCOA. Therefore, the convergence of the RCOA completely depends on the time constant. The convergence rate of the RCOA is proportional to the time constant $\tau$. The expression for the time constant is presented in Eq. (19).

19 $$\tau =\left(a-b\right)\times {r}_{1},$$

where $a$ is constant and is equal to 5, and $b$ and ${r}_{1}$ denote the random numbers between [0,1]. The convergence of the RCOA can be adjusted by adjusting the value of $a$. The optimal value of $a$ is 5, as per the above-all discussions and experimental trials.

The overall response of the RC circuit identifies the position of the best solutions and tries to reach them. The overall response is typically directed by both the transient-state and steady-state responses. To mathematically simulate the RC circuit behaviour, we assume that the steady-state and transient-state have enough information about the possible position of the best solution. Thus, save the optimal solutions (from transient and steady-state) obtained to position update as per the best population position. The mathematical expressions used in this paper are given as follows.

20 $$\overrightarrow{{X}_{1}}=\overrightarrow{{X}_{ts}}-\mathrm{exp}\left(-\frac{t}{\tau }\right),$$

21 $$\overrightarrow{{X}_{2}}=\overrightarrow{{X}_{ss}}\left(1-\mathrm{exp}\left(\frac{t}{\tau }\right)\right),$$

where $\overrightarrow{{X}_{ts}}$ and $\overrightarrow{{X}_{ss}}$ are position vectors (voltage) of the transient-state and steady-state, respectively. Using Eq. (22), it is possible to control the final position (voltage).

22 $$\overrightarrow{X}\left(t+1\right)=\overrightarrow{{X}_{1}}+\overrightarrow{{X}_{2}}.$$

The exploration phase is attained when $\tau <5T$, while the exploitation phase of the RCOA is attained when $\tau \ge 5T$. The pseudocode of the suggested algorithm is illustrated in *Algorithm* 1. The proposed algorithm is very simple, and only Eq. (22) is used to update the position vector and balance between the exploitation and exploitation process.
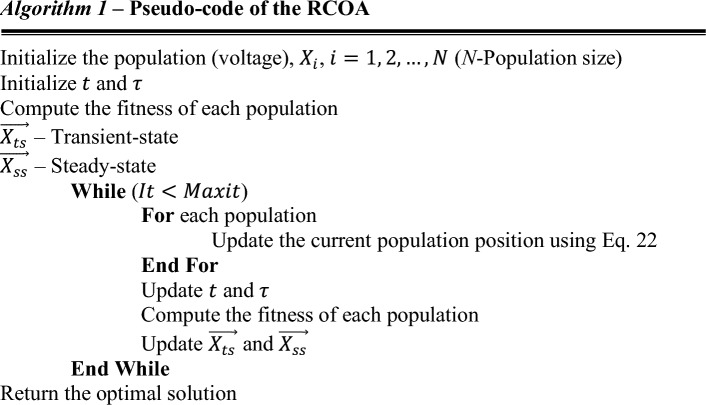


### Implementation procedure of the RCOA

The main motive of this section is to discuss the implementation procedure of the RCOA. The following are the steps to be followed to develop RCOA.

**Step 1:** Initialize the population size ($N$) and the maximum number of iterations ($Maxit$). The initial random solution is generated using Eq. (23) (Initialization phase).

23 $$X=lb+rand\times \left(ub-lb\right),$$

where $X$ denotes the population position, $ub$ denotes the upper bound, $lb$ denotes the lower bound of the decision vectors, and $rand$ denotes the random number between $[\mathrm{0,1}]$.

**Step 2:** Determine the initial capacitor voltage in a *dim*-dimensional search space and the objective function value of the capacitor.

**Step 3:** Sort the best objective function value (${f}_{obj(best)}$) in ascending order and archive it.

**Step 4:** Better exploration and exploitation ability to escape from the local solutions.

**Step 5:** Update the current solution positions as per previous steps, and the voltage of the capacitor (current solution) is updated by Eq. (22).

**Step 6:** Checking for terminating criteria. If the current iteration ($It$) reaches the maximum number of iterations ($Maxit$), the algorithm returns the optimum value; else, go to step 2.

**Figure 10:**
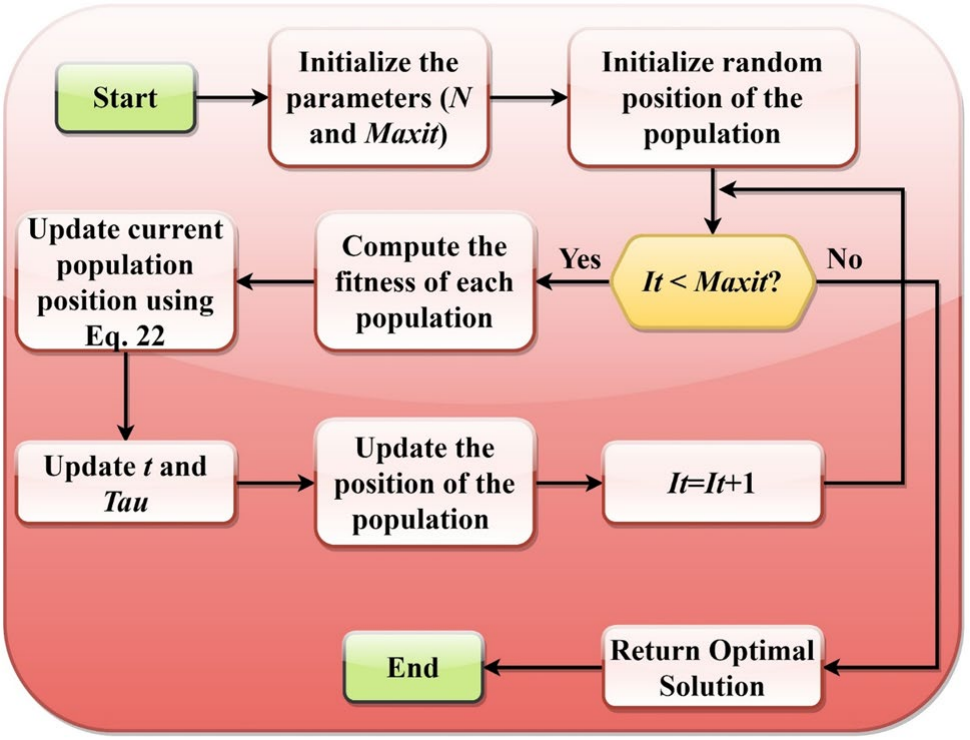
Flowchart of the RCOA.

The flowchart of the proposed RCOA is shown in Fig. 10.

### Computational complexity of RCOA

The following information is provided on the RCOA's time complexity: During the initialization stage, the RCOA necessitates $O(N\times dim)$ time, where $N$ denotes the number of population and $dim$ denotes the dimension of the given problem. The control parameter necessitates $O(N\times dim)$ time. The position update necessitates $O(N\times dim)$ time. The computation of the fitness of each population necessitates $O(N\times dim)$ time.

After careful investigation, it is observed that the time complexity of the RCOA is $O(N\times dim)$ for each iteration. Therefore, the overall time complexity is $O(N\times dim\times Maxit)$, where $Maxit$ denotes the maximum number of iterations.

## Simulation results and discussions

The outcomes of the numerical simulations are thoroughly discussed and analyzed in this section, in addition to the exploitation and exploration of the RCOA. The effectiveness of the RCOA is compared with several competing open-source metaheuristic algorithms, such as MPA, CrSA, MRFO, PSO^93^, JAYA^94^, SCA, and GWO, using a collection of 23 standard benchmark test functions. The proposed algorithm is undoubtedly applicable to any constrained and unconstrained optimization problems. This study has selected 23 unimodal and multimodal benchmark problems with fixed and variable problem dimensions and 8 real-world constrained engineering design problems to prove the same. The proposed algorithm is also provided with a penalty-based constrained handling mechanism. Therefore, the proposed algorithm is not restricted to only the problems selected and discussed in this study. It is suitable for any constrained engineering design problems.

The parameter settings of all algorithms are listed in Table 2. The algorithmic parameters are selected based on the original version. A detailed description of all 23 benchmark test functions can be found in Ref.^95^. The benchmark functions have characteristics such as unimodal (F1–F7), multimodal (F8–F13), and fixed dimension multimodal (F14–F23). The exploitation potential of the RCOA is assessed using unimodal functions. Multimodal functions assess the explorative potential of the RCOA. Fixed-dimension multimodal functions assess the low-dimensional exploration of the RCOA. The population size for all algorithms is selected as 30, and the maximum iterations of all algorithms are 500. The control variables of all chosen algorithms are taken from the literature. For a fair assessment, all algorithms are executed 30 times individually. The simulation experiments were carried out on a laptop operating in Windows 11 with 16 GB memory and a clock frequency of 4.44 GHz. The simulation software called MATLAB was used to code all algorithms that were used in the comparison. MATLAB is a powerful tool that can handle computations involving very large and very small numbers. To be more precise, MATLAB can compute and represent values to a high degree of precision (around 15 decimal places) using double-precision floating-point numbers, the default numerical data type.

**Table 2:** Parameter setting of all algorithms.

Algorithms	Parameters
RCOA	Parameter free
MPA	$FAD=0.2$, and $E=0.5$
CrSA	Parameter free
MRFO	Somersault factor $S=2$
PSO	Inertia weight = 1, Cognitive and social inertia coefficients are equal to 2
JAYA	Parameter free
SCA	$a=2$
GWO	$a= 2 \,\, \mathrm{to} \,\, 0$

### Qualitative analysis

**Figure 11:**
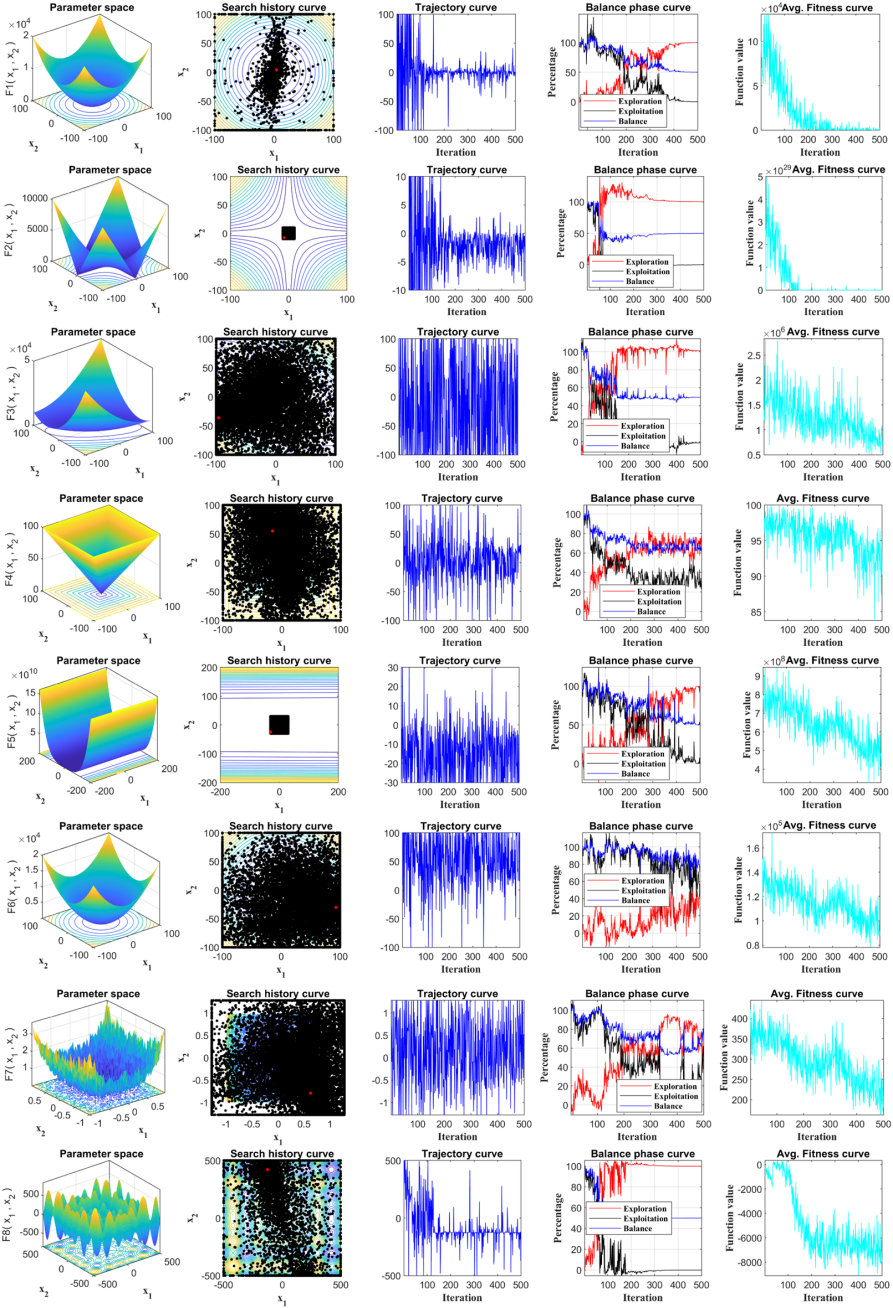

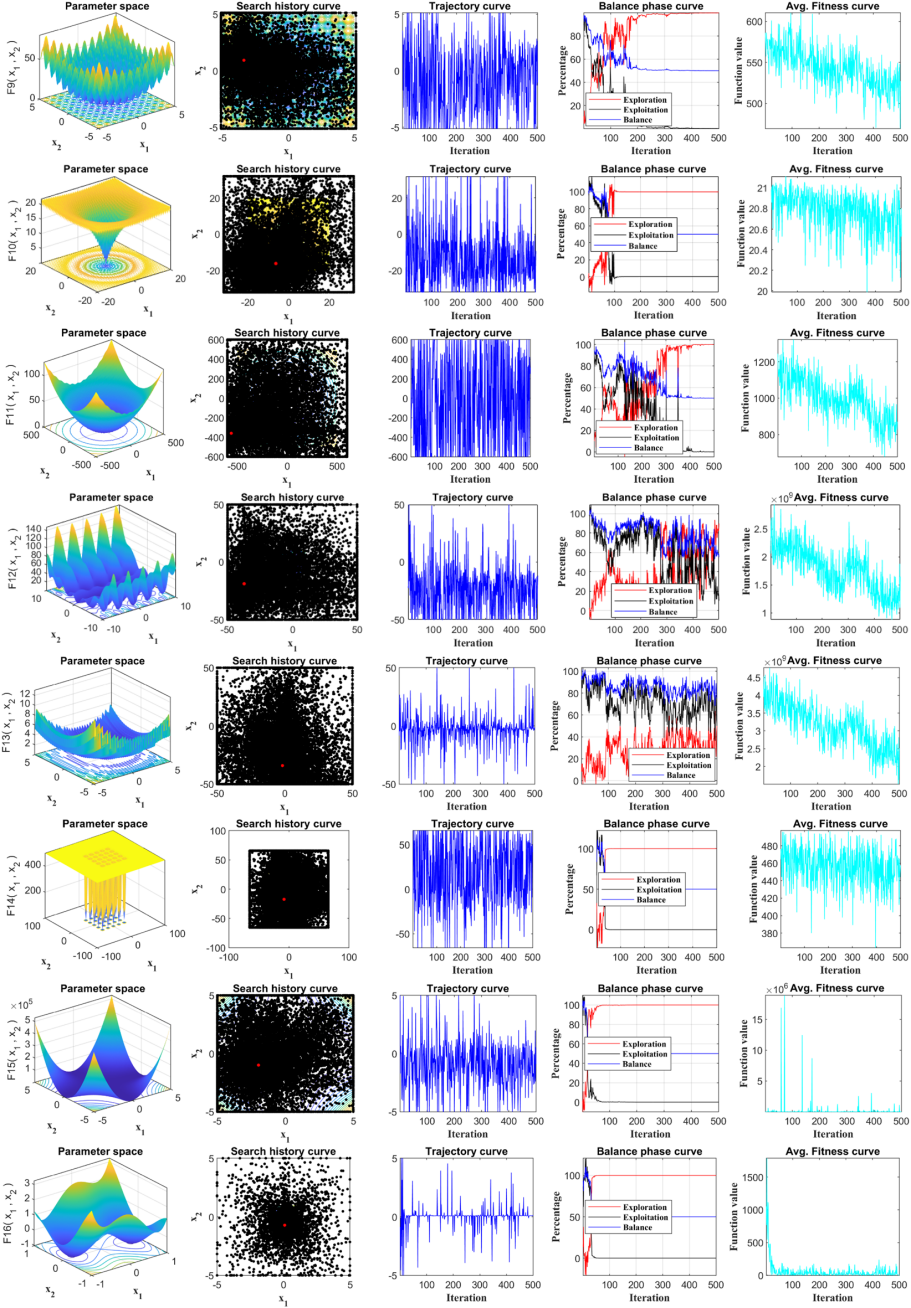

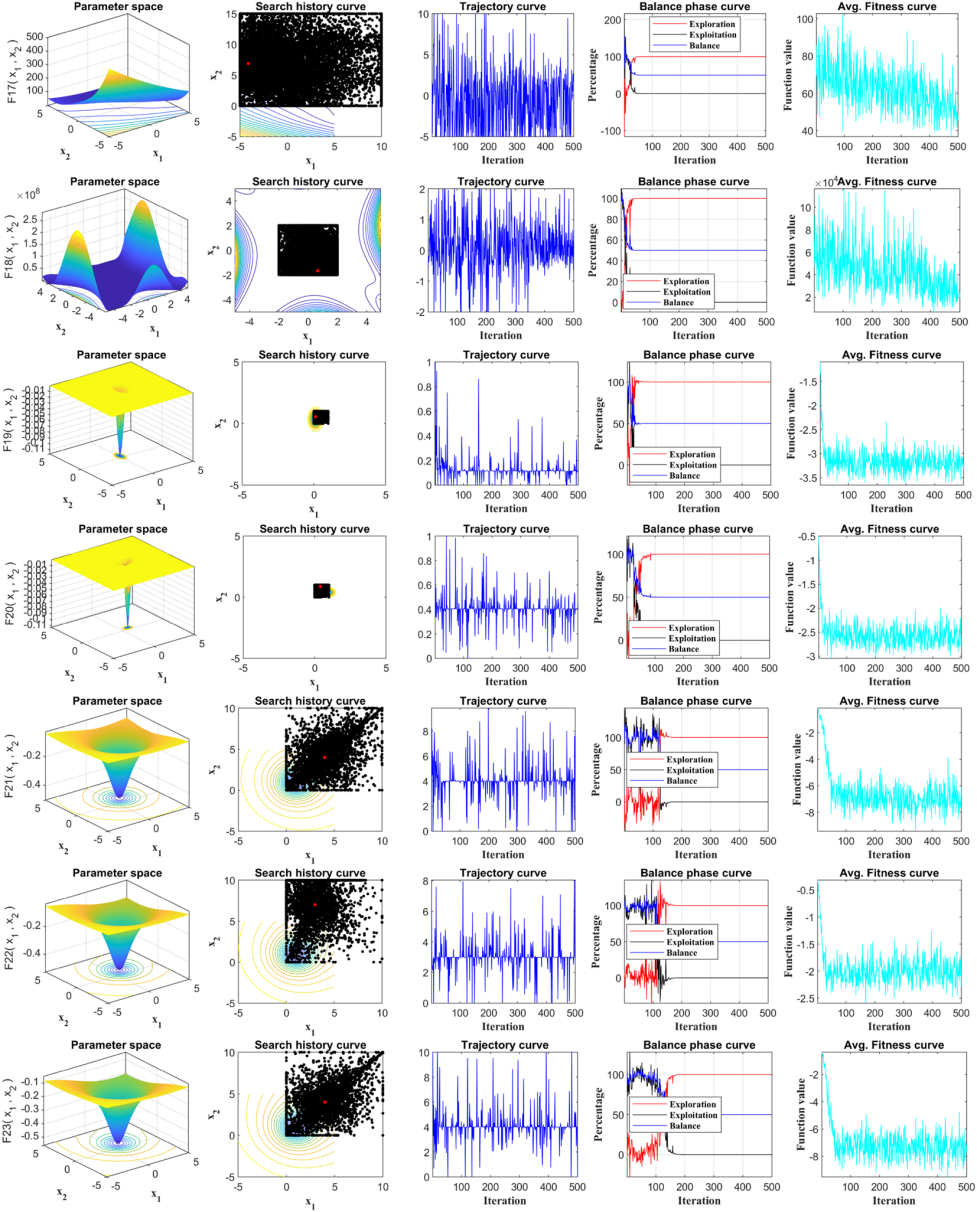
Qualitative analysis of the proposed RCOA on all benchmarks.

In order to evaluate the location and fitness variations, the qualitative data analysis findings of RCOA in dealing with multimodal and unimodal functions are defined in Fig. 11. The graph includes four alarming metrics: search history, trajectory, average fitness, and balance curves. In the iterative procedure, the search history depicts the spread of the population (voltage) and their respective position. The trajectory reveals the features of the population position update in the initial half of the problem dimension. The average fitness variation trend varies with the iterative procedure, as indicated by average fitness. The population in multiple test functions displayed a consistent search trajectory close to the optimum value, as shown by the search history map, effectively discovering dependable search space and displaying high precision. Nevertheless, the population primarily originates in numerous places with local optimums, demonstrating the compromise of the population between multiple local optimums.

As portrayed in Fig. 11, the location curve demonstrates significant variability during the initial iterative process—approximately up to 50% of the exploratory area. This indicates a robust exploration phase wherein the algorithm searches a wide solution space to avoid getting trapped in local optima. As iterations progress, especially if the function under optimization is uniform, the amplitude of the population position gradually diminishes, signifying a transition into the exploitation phase. However, in instances where the function exhibits high variation, the positioning amplitude also notably fluctuates. This variability characteristic can be visually tracked by scrutinizing the mean fitness curve throughout the iterative process. Despite oscillations in the mean fitness curve, there is a general downward trend in the mean fitness values. Notably, the frequency of these oscillations decreases inversely proportional to the number of iterations. This trend allows quicker convergence, indicating the algorithm's efficient navigation towards optimal solutions. Further evaluation of the exploration and exploitation phases greatly contributes to understanding the RCOA's performance. We utilized all benchmark functions to delve into two vital features—exploration and exploitation—which are integral to assessing the competency of the RCOA. Visual evidence of a balanced interaction between the exploration and exploitation phases is demonstrated in Fig. 11. The ratios of exploitation and exploration represent this balance within the search bounds. As the curves in Fig. 11 reveal, the RCOA successfully maintains an equilibrium between exploitation and exploration for most of the search duration. This equilibrium is crucial for an optimization algorithm, as it signifies its ability to explore new areas and exploit known good areas in the solution space, ensuring comprehensive and effective optimization.

Standard deviation (STD) and average (Avg.) results were used to examine the outcome of the experiment for the aim of determining overall significance. Table 3 collates the statistical results of applying selected algorithms on 23 standard benchmark functions as presented in the study. A subsequent comparative analysis of these results highlighted the distinct performance superiority of the RCOA. The RCOA found a larger fraction of optimal values, specifically 19 out of 23, surpassing all its peers in this comparison. Consequently, the RCOA emerged as the top-ranking algorithm, outperforming others like the GWO and MRFO algorithms, which came in second place. It is worth noting that while some algorithms, such as F23, exhibit favourable results, they can paradoxically yield negative results for other functions. This suggests a lack of consistency and balance in these algorithms' exploration and exploitation phases. However, as presented in Table 3, the RCOA demonstrated commendable outcomes across most functions. This performance profile underscores the RCOA's ability to strike an effective equilibrium between the exploration and exploitation phases—an integral aspect of successful optimization algorithms. Additionally, F8, known as a challenging test function, also succumbed to the RCOA's efficient problem-solving capabilities. Not only was the RCOA able to solve it, but it also identified the optimal solution in the shortest possible time. This indicates the RCOA's ability to handle complex optimization problems, further cementing its superiority and effectiveness in the mathematical optimization domain. The better results are highlighted with boldfaces, which applies to all tables.

**Table 3:** Statistical values obtained by all algorithms for 23 benchmark test suite.

		RCOA	MPA	MRFO	CrSA	PSO	JAYA	GWO	SCA
F1	Avg	**3.18E−101**	6.62E−50	4.71E−19	1.40E + 00	1.10E + 03	6.19E + 04	1.27E−27	1.66E + 01
	STD	**1.32E−95**	5.68E−50	2.01E−16	4.86E−01	4.72E + 03	5.06E + 03	2.85E−27	3.42E + 01
F2	Avg	**4.98E−55**	2.45E−29	1.67E−09	6.59E−01	2.65E + 01	6.82E + 09	7.40E−17	6.49E−04
	STD	**9.36E−53**	2.42E−29	7.03E−09	1.07E−01	8.84E + 00	6.54E + 10	4.93E−17	2.07E−02
F3	Avg	**8.31E−84**	8.17E−16	3.36E−19	1.34E + 02	2.06E + 04	1.65E + 05	5.23E−07	6.29E + 03
	STD	**3.08E−78**	1.81E−13	3.58E−15	8.26E + 01	5.06E + 03	1.88E + 04	2.52E−03	6.72E + 03
F4	Avg	**2.20E−54**	4.85E−13	5.07E−12	2.56E + 00	6.66E + 01	8.90E + 01	4.20E−07	3.32E + 01
	STD	**5.91E−49**	4.02E−12	1.04E−10	1.80E + 00	9.42E + 00	3.40E + 00	1.29E−07	1.06E + 01
F5	Avg	**2.50E−03**	2.86E + 01	2.87E + 01	5.78E + 01	5.37E + 05	2.51E + 08	2.62E + 01	1.02E + 04
	STD	**5.58E−03**	3.46E−01	8.20E−02	5.52E + 01	2.76E + 07	3.48E + 07	7.92E−01	2.24E + 04
F6	Avg	**7.37E−05**	4.03E + 00	1.71E + 00	1.21E + 00	3.96E + 03	6.18E + 04	1.00E + 00	2.40E + 01
	STD	**1.75E−04**	7.06E−01	1.07E + 00	1.57E−01	4.34E + 03	3.30E + 03	2.66E−01	1.09E + 01
F7	Avg	**1.94E−04**	1.79E−03	8.69E−04	3.91E−02	6.98E−01	1.08E + 02	1.53E−03	8.78E−02
	STD	**1.67E−04**	1.21E−03	6.27E−04	1.19E−02	7.08E + 00	1.26E + 01	1.46E−03	7.19E−02
F8	Avg	**−1.26E + 4**	−5.59E + 3	−5.26E + 3	−7.95E + 3	−9.29E + 3	−2.42E + 3	−6.29E + 3	−3.75E + 3
	STD	**3.35E−01**	7.88E + 02	8.51E + 01	4.13E + 02	8.95E + 02	5.99E + 02	3.22E + 02	2.28E + 02
F9	Avg	**0.00E + 00**	1.57E + 01	**0.00E + 00**	1.46E + 02	1.38E + 02	4.20E + 02	5.68E−13	3.60E + 01
	STD	**0.00E + 00**	8.71E + 00	**0.00E + 00**	1.48E + 01	2.29E + 01	1.62E + 01	1.97E + 00	2.85E + 01
F10	Avg	**8.88E−16**	1.51E−14	2.81E−11	2.15E + 00	2.00E + 01	2.00E + 01	1.00E−13	5.28E + 00
	STD	**0.00E + 00**	3.18E−15	5.00E−11	3.72E−01	1.28E−03	3.61E−01	1.70E−14	9.50E + 00
F11	Avg	**0.00E + 00**	7.60E−03	**0.00E + 00**	8.72E−01	1.28E + 01	5.87E + 02	**0.00E + 00**	1.04E + 00
	STD	**0.00E + 00**	5.90E−03	2.09E−15	1.03E−01	3.72E + 01	8.19E + 01	5.35E−03	2.27E−01
F12	Avg	**1.75E−06**	5.12E−01	1.47E−01	1.86E + 00	7.25E + 05	5.75E + 08	3.37E−02	7.85E + 00
	STD	**7.22E−06**	3.41E−01	4.82E−02	8.33E−01	3.52E + 06	1.14E + 08	1.48E−02	9.44E + 01
F13	Avg	**4.43E−05**	1.70E + 00	1.05E + 00	2.22E−01	1.22E + 06	1.02E + 09	8.06E−01	3.81E + 03
	STD	**7.13E−05**	2.51E−01	2.57E−01	1.30E−01	2.77E + 06	2.91E + 08	2.20E−01	2.20E + 06
F14	Avg	**9.98E−01**	1.08E + 01	**9.98E−01**	**9.98E−01**	1.46E + 00	4.02E + 00	1.08E + 01	**9.98E−01**
	STD	4.45E−01	5.60E + 00	1.38E−08	**2.52E−11**	2.16E + 00	5.94E + 00	5.60E + 00	8.87E−01
F15	Avg	**3.59E−04**	5.19E−04	5.13E−04	7.79E−04	7.83E−04	3.18E−03	2.04E−02	1.29E−03
	STD	**2.06E−05**	3.82E−02	1.01E−04	9.58E−03	9.52E−03	1.67E−02	1.09E−02	4.71E−04
F16	Avg	**−1.03E + 0**	**−1.03E + 0**	**−1.03E + 0**	**−1.03E + 0**	**−1.03E + 0**	−9.09E−1	**−1.03E + 0**	**−1.03E + 0**
	STD	**1.32E−09**	1.73E−02	1.82E−07	2.63E−07	0.00E + 00	1.67E−01	1.08E−08	8.51E−05
F17	Avg	**3.98E−01**	**3.98E−01**	**3.98E−01**	**3.98E−01**	**3.98E−01**	4.04E−01	**3.98E−01**	**3.98E−01**
	STD	1.14E−05	8.82E−02	5.33E−05	3.57E−07	**0.00E + 00**	9.21E−02	2.31E−06	2.50E−03
F18	Avg	**3.00E + 00**	**3.00E + 00**	**3.00E + 00**	**3.00E + 00**	**3.00E + 00**	6.81E + 00	**3.00E + 00**	**3.00E + 00**
	STD	3.68E−08	1.16E−06	1.17E−04	1.82E−06	**7.69E−16**	3.53E + 01	6.61E−05	7.64E−05
F19	Avg	−3.8620	**−3.8628**	−3.8625	**−3.8628**	**−3.8628**	−3.6665	−3.8627	−3.8543
	STD	2.20E−03	5.37E−06	1.39E−04	**4.94E−07**	4.32E−03	2.60E−01	3.29E−03	2.82E−03
F20	Avg	−3.2033	−3.3217	−3.3090	−3.2023	−3.1299	−2.1002	**−3.3220**	−3.0130
	STD	5.75E−02	5.34E−02	7.26E−03	6.62E−02	1.15E−01	4.88E−01	**1.18E−05**	5.18E−02
F21	Avg	−5.0477	−10.1198	−10.0808	−5.1007	−2.6829	−0.5977	**−10.1507**	−0.8783
	STD	**8.75E−03**	2.77E + 00	5.64E−02	3.38E + 00	3.26E + 00	1.43E−01	2.26E + 00	1.80E + 00
F22	Avg	**−10.4006**	−10.3919	−5.0857	−10.4025	−3.7243	−0.6350	−10.2827	−2.8679
	STD	**1.53E−03**	2.36E + 00	3.78E−03	3.42E + 00	3.85E + 00	1.08E−01	5.18E−02	1.94E + 00
F23	Avg	−10.5352	−5.1261	−10.4346	**−10.5361**	−5.1285	−0.8059	−10.5288	−4.1682
	STD	**5.76E−04**	1.61E−03	2.61E−02	3.69E + 00	4.10E + 00	2.02E−01	8.55E−03	9.07E−01

Significant values are in bold.

Table 4 shows that the performance of the RCOA is evaluated using a statistical Wilcoxon signed-rank test (WSRT) at a 5% significant significance level. Table 4 shows the WSRT results, which calculated the rank of all algorithms for each benchmark function. The summation of all rankings for all 23 test functions was then computed, as shown in Table 4. These findings indicated that the RCOA came out on top when compared to the other algorithms in the test group. Additionally, the *p*-value for each benchmark function is determined, and the null hypothesis (which claims that there is no difference among algorithms) is discarded since all *p*-values are less than the statistically significant level. Table 4 also compares the runtime (RT) of the RCOA with other algorithms. Because of its simplicity should go without saying that the JAYA algorithm has a low RT value with a total runtime (TRT) of 0.35, followed by the RCOA with 1.92 TRT, which has the second shortest RT. The MRFO algorithm, conversely, has an extended RT with 26.957 TRT. The rank (R) has been allocated for all algorithms based on TRT values of benchmark functions. The JAYA algorithm stood first based on TRT values, followed by RCOA, GWO, SCA, PSO, CrSA, MPA, and MRFO.

**Table 4:** RT values of all algorithms for 23 benchmark functions.

	RCOA	MPA	MRFO	CrSA	PSO	JAYA	SCA	GWO	*p*-Value
F1	0.106	0.169	1.656	0.272	0.125	0.009	0.116	0.109	7.937E−03
F2	0.050	0.088	1.081	0.094	0.075	0.008	0.069	0.069	7.937E−03
F3	0.159	0.356	1.525	0.194	0.166	0.003	0.163	0.159	7.937E−03
F4	0.044	0.078	0.903	0.094	0.066	0.003	0.053	0.063	7.937E−03
F5	0.063	0.159	0.956	0.119	0.078	0.003	0.069	0.072	7.937E−03
F6	0.045	0.109	0.950	0.097	0.069	0.003	0.066	0.066	7.937E−03
F7	0.106	0.216	1.238	0.163	0.116	0.048	0.128	0.119	2.041E−02
F8	0.094	0.169	1.109	0.072	0.109	0.070	0.100	0.097	7.937E−03
F9	0.050	0.103	0.928	0.106	0.072	0.010	0.063	0.063	1.497E−01
F10	0.069	0.122	0.959	0.109	0.075	0.011	0.066	0.056	7.937E−03
F11	0.075	0.141	1.056	0.119	0.075	0.015	0.084	0.072	1.723E−01
F12	0.213	0.506	1.756	0.244	0.216	0.006	0.225	0.216	7.937E−03
F13	0.206	0.491	1.631	0.250	0.216	0.015	0.225	0.203	7.937E−03
F14	0.313	0.866	2.341	0.334	0.319	0.016	0.328	0.350	4.717E−02
F15	0.019	0.091	0.888	0.044	0.031	0.010	0.031	0.034	4.531E−02
F16	0.028	0.081	0.791	0.034	0.028	0.009	0.019	0.031	7.937E−03
F17	0.044	0.125	1.259	0.088	0.041	0.007	0.053	0.044	2.902E−01
F18	0.016	0.066	0.738	0.031	0.025	0.010	0.019	0.019	7.937E−03
F19	0.031	0.119	1.022	0.078	0.031	0.014	0.031	0.031	2.449E−01
F20	0.047	0.122	1.078	0.053	0.044	0.026	0.031	0.041	2.483E−01
F21	0.041	0.156	0.969	0.056	0.041	0.017	0.041	0.038	1.179E−01
F22	0.050	0.166	1.009	0.056	0.044	0.003	0.044	0.047	1.259E−01
F23	0.053	0.200	1.113	0.069	0.050	0.003	0.053	0.056	1.259E−01
TRT	1.920	4.697	26.957	2.776	2.110	0.315	2.075	2.054	–
R	2	7	8	6	5	1	4	3	–

**Figure 12:**
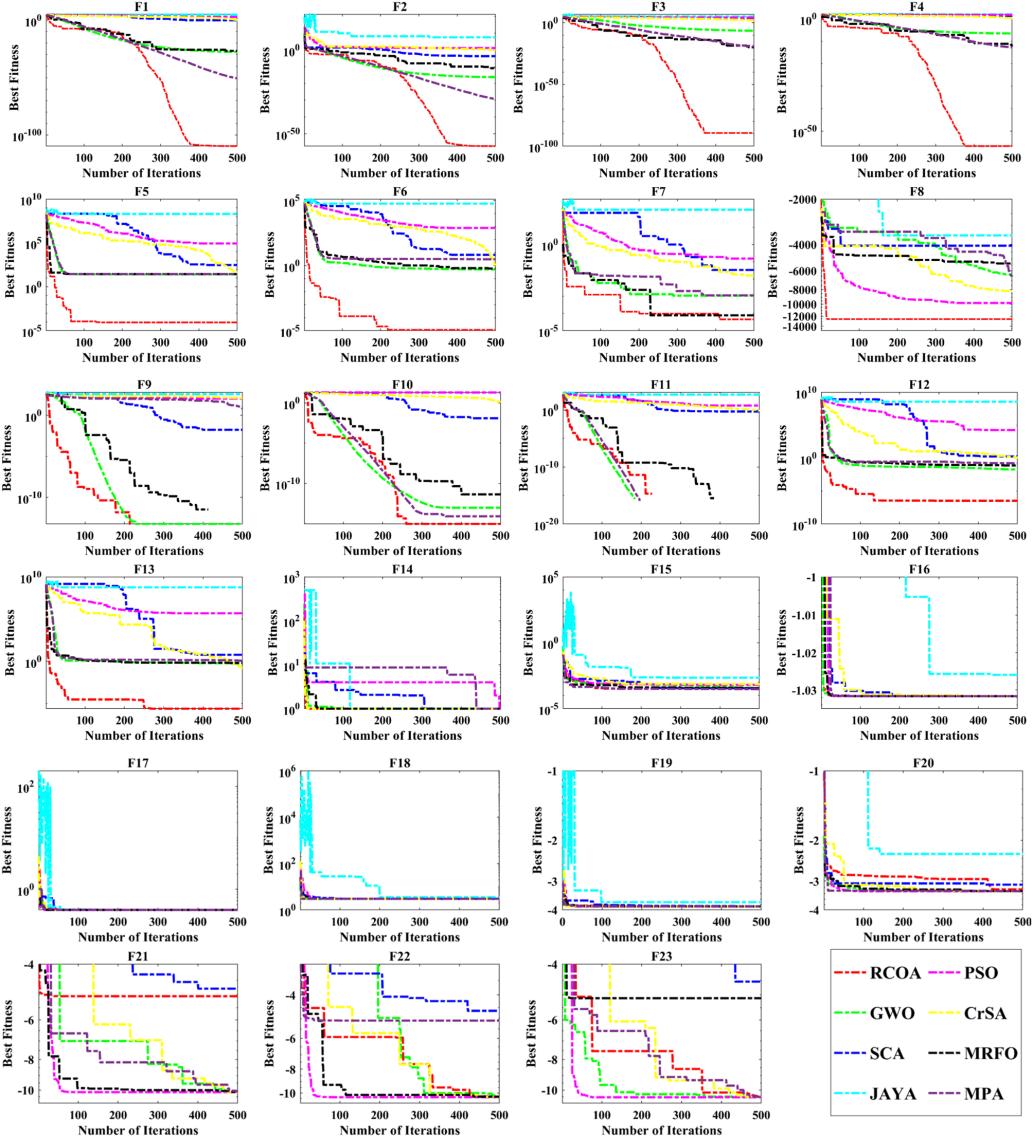
Averaged convergence curves of all algorithms on all benchmark functions.

Figure 12 showcases the significant advantage of RCOA in terms of convergence speed compared to other algorithms in the field. The convergence graph reflects the optimal fitness value during the iteration phase, with the convergence curve further illustrating how the population's ideal fitness value—represented here as voltage—enhances over time with respect to the mean fitness. The rate of convergence and the precise moment when the algorithm shifts from the exploration phase to the exploitation phase can be inferred from the downtrend of the curve. In most of these graphical representations, the time transition proves beneficial to the RCOA. It assists the algorithm in effectively exploring and exploiting the domain under consideration, thereby augmenting its performance. Towards the final iterations, the convergence curves in these results exhibit a stable trend, denoting that the conditions for convergence have been met. This stability also signals the reliability of the RCOA during the problem-solving process. Built on solid foundations, the RCOA is able to locate the global optimum for unimodal functions. However, the true power and utility of the RCOA emerge when it is employed for more complex multimodal functions. In these complex scenarios, the nuanced performance of the algorithm and its procedures comes to the fore, illustrating both its value and the benefits it provides in mathematical optimization.

**Figure 13:**
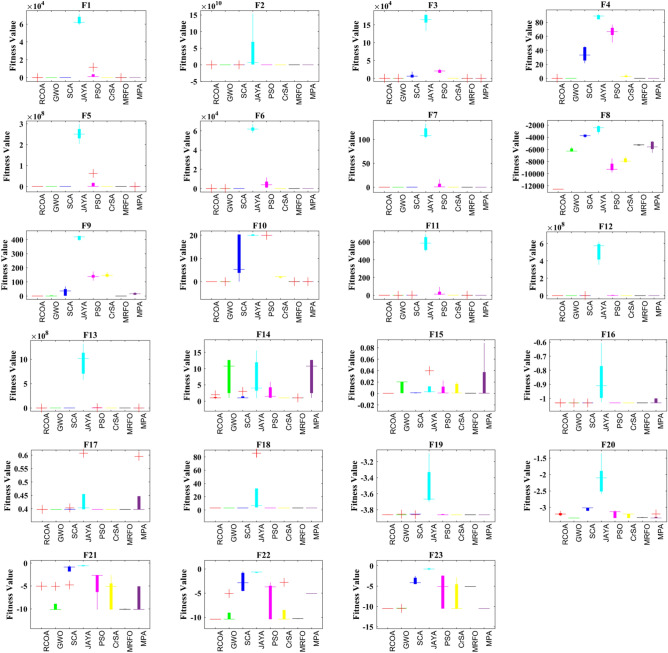
Boxplot obtained by all algorithms on 23 standard test functions.

However, each selected method's reliability is assessed using the boxplot. In Fig. 13, all selected algorithms are boxplot-evaluated against a set of 23 benchmark functions. Graphical representations are provided to prove that the suggested RCOA is more reliable than any other algorithms considered.

### Importance of a superiority assessment

The WSRT method has been used to determine whether or not RCOA offers evident benefits over pairwise comparison in terms of accuracy. If the *p*-value generated by the assessment is less than the 5% level of significance, it indicates that the algorithm's results in pairwise comparison are statistically superior. Other than that, the differences between the two competitors are believed to be inconsequential from a statistical standpoint. The real statistical significance of the integrated pairwise comparison is presented in Eq. (24) to draw more complete conclusions.

24 $$p=1-\prod_{i=1}^{j-1}1-{p}_{h1}.$$

Table 3 shows the *p*-value obtained using Eq. (24), which shows that the *p*-values in F1-8, F10, F12-F16, and F18 were less than 5% compared to all selected algorithms. Consequently, as compared to other algorithms, RCOA performs significantly better in such functions than the latter. When comparing algorithms, although pairwise comparisons can be employed, the error rate produced during the test cannot be adjusted, and the selection of algorithms in numerous analyses can significantly impact the outcome of the study. Multiple comparison procedures are utilized to change the error rate to decrease the impact of algorithm selection for each result set.

When performing numerous comparisons, the first step is to determine whether the results generated by the algorithm are equivalent. When an inequity occurs, a post-hoc analysis should be performed to determine which algorithms have statistically significant differences. As a result, Friedman's rank test (FRT) was used, a non-parametric test. Table 5 depicts FRT values obtained for all 23 benchmark functions. The average FRT (AFRT) values are provided in Table 5. Based on the AFRT values, the rankings are provided. The RCOA took the lead with a 1.935 AFRT value, preceded by GWO, MRFO, MPA, CrSA, PSO, SCA, and JAYA.

**Table 5:** FRT values of all algorithms for 23 benchmark functions.

	RCOA	MPA	MRFO	CrSA	PSO	JAYA	SCA	GWO
F1	**1**	2	3.8	5.2	7	8	5.8	3.2
F2	**1**	2	4	6	7	8	5	3
F3	**1**	3	2	5	7	8	6	4
F4	**1**	2.2	2.8	5	7	8	6	4
F5	**1**	3.2	3.8	5	7	8	6	2
F6	**1**	5	3.2	3	7	8	6	2.8
F7	**1.2**	3.6	1.8	5.2	7	8	5.8	3.4
F8	**1**	5	5.6	2.8	2.2	8	7	4.4
F9	**1.5**	4.4	**1.5**	6.6	6.4	8	4.4	3.2
F10	**1**	2	4	5.2	7.2	7.2	6.4	3
F11	**2**	3.2	2.3	5.2	7	8	5.8	2.5
F12	**1**	3.8	3.2	5.4	7	8	5.6	2
F13	**1**	4.8	3.8	2	6.6	8	6.4	3.4
F14	**1.8**	6.8	3	**1.8**	5.8	6.2	4.2	6.4
F15	**1.6**	4.6	2.8	5.4	5.6	6.4	4.4	5.2
F16	2	5.2	5.6	4.6	**1**	8	6.6	3
F17	3.2	5	6	2.8	**1**	7.2	7.2	3.6
F18	2	3.6	6	3.8	**1**	8	6.2	5.4
F19	5	2.8	4.4	**1.6**	3.2	8	6.8	4.2
F20	4.8	3.2	3.6	3.6	4	8	7	**1.8**
F21	5.2	2.8	3	3.6	4.8	7.8	7	**1.8**
F22	**2.2**	3.4	5.2	2.4	4	7.8	7	4
F23	**2**	5.4	4	2.8	4.2	8	6.6	3
AFRT	**1.935**	3.783	3.713	4.087	5.217	7.765	6.052	3.448
R	**1**	4	3	5	6	8	7	2

Significant values are in bold.

### Sensitivity analysis

**Figure 14:**
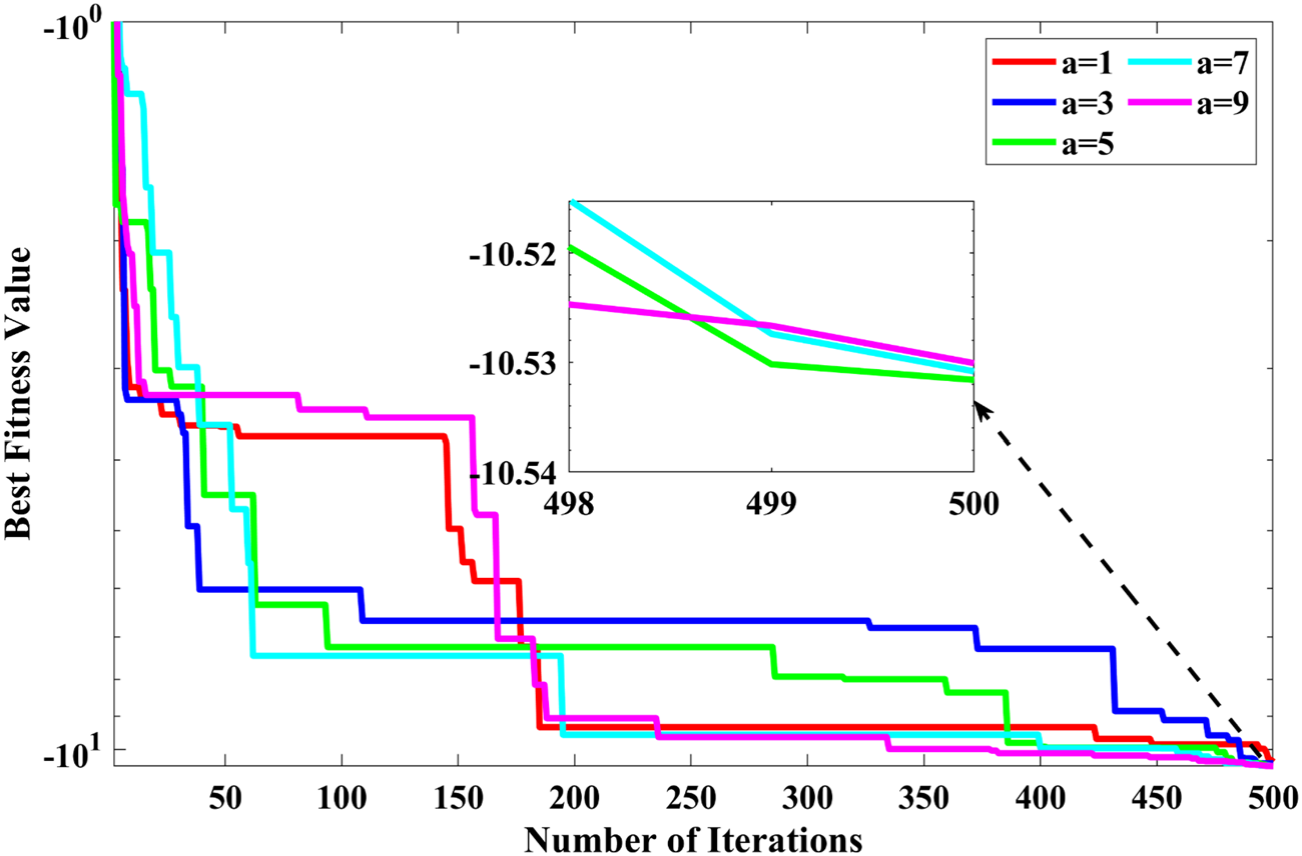
Convergence curve obtained by RCOA for F23 function with diverse values of $a$.

The parameter sensitivity test was included in this subsection to investigate the effects of the algorithm's population size ($N$) and parameter $a$ on the algorithm. The parameter $a$ has different ranges, such as 1, 2, 3, 4, and 5. The population sizes were selected as 10, 20, 30, 40, and 50. The number of iterations was set at 500. The function F13 has been selected to demonstrate the parameter sensitivity. The convergence curves of function F13 for different values of $a$ have been plotted and shown in Fig. 14. It is seen from Fig. 14 that the RCOA is superior when the value of $a$ is 5. This has already been discussed in the mathematical modelling of the proposed algorithm. If the value of $a$ is greater than 5, the RCOA gives the same fitness value. But, if the value of $a$ is less than 5, the fitness value may be less than the best optimal. Therefore, in this paper, the value of $a$ is selected as 5 for test cases.

**Figure 15:**
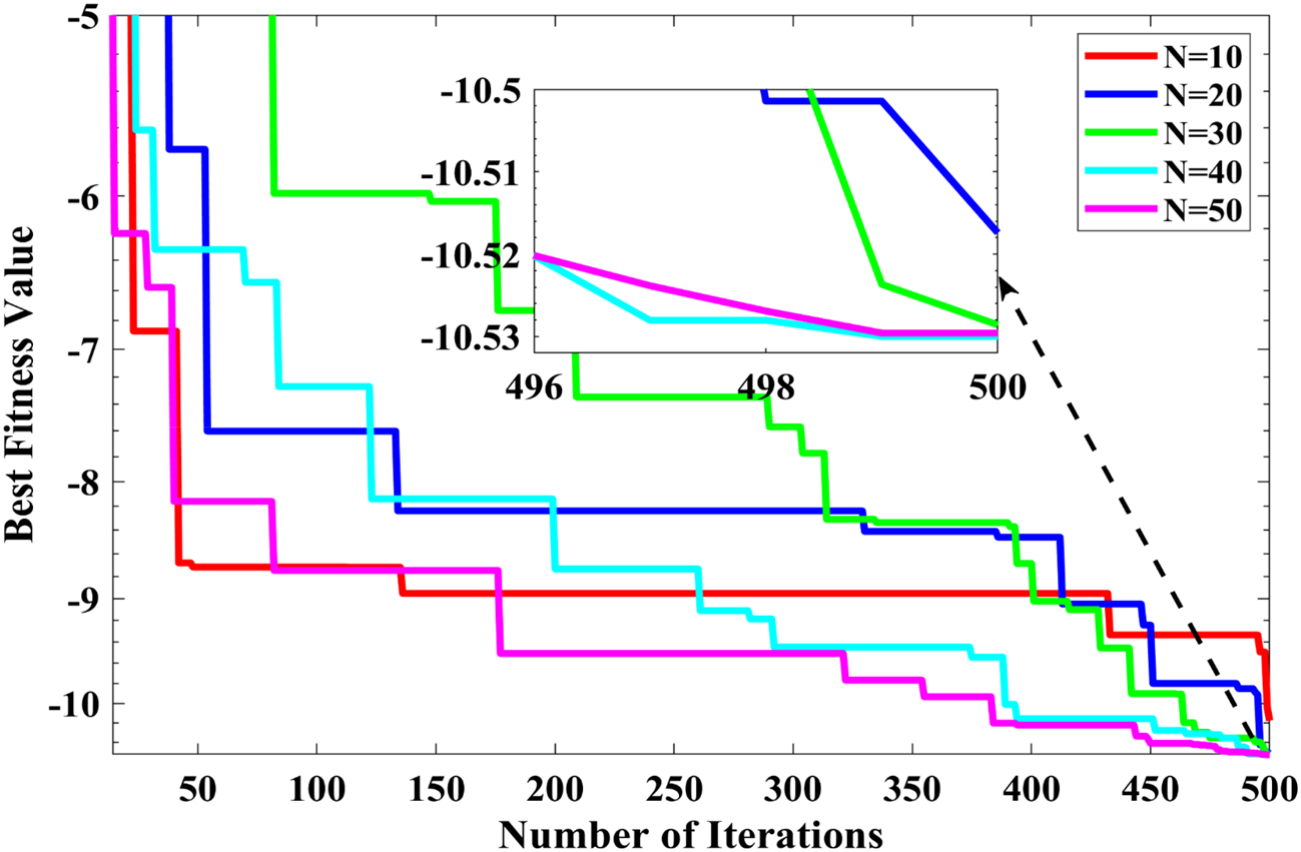
Convergence curve obtained by RCOA for F23 function with different values of $N$.

We selected function F23 to investigate the impact of the population size on the algorithm performance. Based on the previous discussion, the value of $a$ is fixed at 5. This parameter was chosen to investigate the synergistic effect of the population size on the algorithm. Figure 15 shows the convergence curve for the F23 function by RCOA with different values of population size. It can be seen explicitly that the mean value becomes better as the population size increases. The rationale for this is that increasing the number of populations increases search efficiency. Nevertheless, because the global approximative optimal solution was obtained, the results did not improve as the size of the population increased. According to the specific problems individuals attempt to rectify, researchers can decide on an adequate population size. However, this paper selected the population size for the benchmark functions as 30.

## Real-world engineering design optimization problems

This section comprehensively discusses the application of the proposed RCOA for solving real-world engineering design problems. The problems solved in this section are categorized as constraint engineering design problems. Firstly, the fitness functions and different constraints of each problem are provided. All the selected problems are solved by applying the algorithms, including the proposed RCOA. Finally, a thorough analysis is conducted depending on the outcomes of all chosen algorithms.

### Constraint handling mechanism (CHM)

Most engineering design problems aim to find the best solution under particular circumstances, typically based on constrained resources, design guidelines, and safety criteria. Such special restrictions are referred to as constraints, and the primary goal of optimization is to develop a reasonable and efficient solution ^96^. The optimization issue can be expressed in the following way:

25 $$\mathrm{Minimize} \to f\left(X\right), X=\left({x}_{1},{x}_{2},\dots ,{x}_{dim}\right)$$

Subjected to:

26 $$\left.\begin{array}{c}{g}_{i}\left(X\right)\le 0, i=1, 2, \dots , {N}_{g}\\ {h}_{j}\left(X\right)=0, j=1, 2, \dots , {N}_{h}\\ {lb}_{k}\le {x}_{k}\le {ub}_{k}, k=1, 2, \dots ,dim\end{array}\right\},$$

where $f\left(X\right)$ denotes fitness/objective function, $X$ denotes the solution vector, and ${h}_{j}\left(X\right)$ and ${g}_{i}\left(X\right)$ denote equality and inequality constraints, respectively. ${N}_{h}$ denotes the number of equality constraints, ${N}_{g}$ denotes the number of inequality constraints, $dim$ denotes the problem dimension, and ${ub}_{k}$ and ${lb}_{k}$ denote the maximum and minimum bounds for the $k$th dimension/variable, respectively. The idea to emphasize would be that a viable solution meets all of the constraints. The converse is true for weak solutions, which fail to meet a minimum of one constraint^97^. In most cases, the equality constraints ${h}_{j}\left(X\right)=0$ are replaced with an inequality constraint $\left|{h}_{j}\left(X\right)-\varepsilon \right|\le 0$ to handle the constraint problems, in which $\varepsilon$ denotes a penalty factor. An alternative method for solving the equality constraints is to substitute the equality constraints with two inequality constraints ${h}_{j}\left(X\right)\ge 0$ and ${h}_{j}\left(X\right)\le 0$. This method aids in fast convergence to its optimal state, converting the constraints into inequality constraints.

All the optimization algorithms are unable to tackle constraint problems straightforwardly. As a result, giving them access to an extra mechanism for coping with constraints is necessary. The constraint-handling techniques group of approaches was specifically developed for this concept and is referred to as CHMs. The CHMs allow the optimization techniques to deal with the governing equations simultaneously without sacrificing performance. In general, constraint handling mechanisms are classified into five groups^98^: (i) penalty functions, (ii) repair algorithms, (iii) parting of constraints and objectives, (iv) specific operators and representations, and (v) hybrid approaches. The first method, which is based on the penalty function, is a simple and well-established method for dealing with limitations. After adding a penalty to the fitness function, it converts a constraint problem into an unconstraint optimization problem, resulting in a win–win situation. A penalty function can be written in the following way:

27 $$F\left(X\right)=f\left(X\right)+P\left(X\right),$$

where $F\left(X\right)$ denotes the transformed objective function, $f\left(X\right)$ denotes the actual fitness function, and $P\left(X\right)$ denotes the penalty function that signifies the constraint violations and is represented as follows ^97^:

28 $$P\left(X\right)=\sum_{i=1}^{{N}_{g}}{\delta }_{i}\times \mathrm{max}\left(0,{g}_{i}(X)\right)+\sum_{j=1}^{{N}_{h}}{\rho }_{j}\times \mathrm{max}\left(0,\left|{h}_{j}\left(X\right)\right|-\varepsilon \right),$$

where $\mathrm{max}\left(0,{g}_{i}(X)\right)$ and $\mathrm{max}\left(0,\left|{h}_{i}\left(X\right)\right|-\varepsilon \right)$ denote the solution violations as per $i$th inequality constraint and $j$ ^th^ equality constraint, respectively. In addition, ${\delta }_{i}$ and ${\rho }_{j}$ denote penalty factors. The degree of penalty factors impacts the quality of solutions, and the appropriate penalty factors vary depending on the situation. Constraint problems can be resolved using metaheuristic approaches, and CHM is used for recognizing the possible search space for limitations. Once a viable location has been identified, an algorithm should attempt to identify an optimal or near-optimal solution inside that zone. As a result, in every iteration of an algorithm, the population fitness is assessed as per the objective function and constraints, and the next iteration is formed based on the objective function. The algorithm would determine the problem's search location based on the objective function value of the existing population. Therefore, this paper uses the penalty function approach to solve all selected constraint engineering design optimization problems.

### Real-world optimization problems

This sub-section comprehensively analyzes the performance of the proposed RCOA and other selected algorithms on 10 engineering design optimization problems.

#### Speed reducer design optimization (SRDO) problem

**Figure 16:**
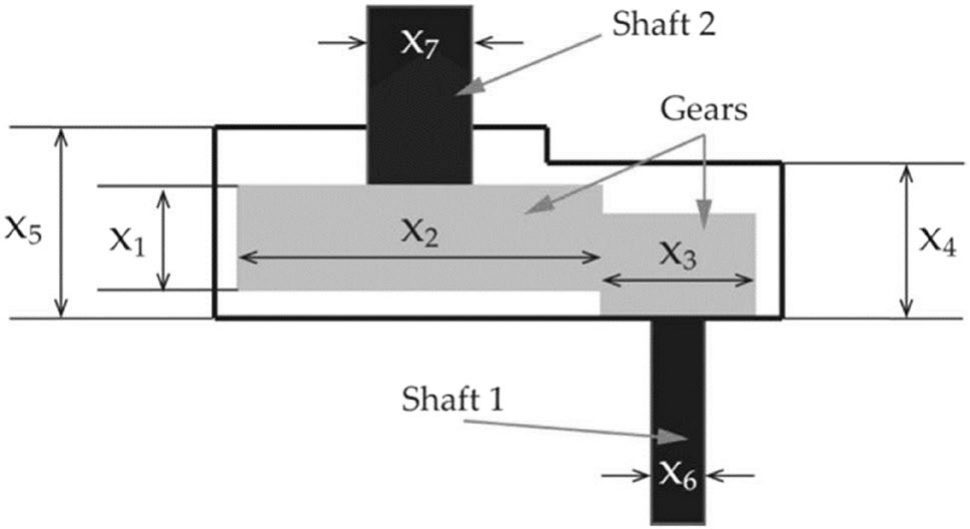
Internal structure of speed reducer.

Figure 16 depicts the structure of the SRDO problem and its components. Because of its defined constraints and complex search space, this design engineering challenge is regarded to be one of the most difficult problems to solve. The fitness function of the SRDO problem, as presented in Eq. (29), is to reduce the gearbox weight to its smallest possible value while taking into account 11 distinct constraints. There are also numerical constraints on the shaft stress, surface stress, shaft transverse deflections, and bending stress that must be satisfied.

29 $$\text{Min: }f\left(\overrightarrow{x}\right)=0.7854{x}_{1}{x}_{2}^{2}\left(3.3333{x}_{3}^{2}+14.9334{x}_{3}-43.093\right)-1.508\left({x}_{6}^{2}+{x}_{7}^{2}\right)+7.4777\left({x}_{6}^{3}+{x}_{7}^{3}\right)+0.7854\left({x}_{4}{x}_{6}^{2}+{x}_{5}{x}_{7}^{2}\right),$$

Subjected to:

$${g}_{1}\left(\overrightarrow{x}\right)=\frac{27}{{x}_{1}{x}_{2}^{2}{x}_{3}}\le 0$$

$${g}_{2}\left(\overrightarrow{x}\right)=\frac{397.5}{{x}_{1}{x}_{2}^{2}{x}_{3}}\le 0$$

$${g}_{3}\left(\overrightarrow{x}\right)=\frac{1.93{x}_{4}^{3}}{{x}_{2}{x}_{6}^{2}{x}_{3}}\le 0$$

$${g}_{4}\left(\overrightarrow{x}\right)=\frac{1.93{x}_{5}^{3}}{{x}_{2}{x}_{7}^{2}{x}_{3}}\le 0$$

$${g}_{5}\left(\overrightarrow{x}\right)=\frac{1.0}{110{x}_{6}^{3}}\sqrt{{\left(\frac{745{x}_{4}}{{x}_{2}{x}_{3}}\right)}^{2}} +16.9\times {10}^{6}-1\le 0$$

$${g}_{6}\left(\overrightarrow{x}\right)=\frac{1.0}{85{x}_{7}^{3}}\sqrt{{\left(\frac{745{x}_{5}}{{x}_{2}{x}_{3}}\right)}^{2}} +1.579\times {10}^{6}-1\le 0$$

$${g}_{8}\left(\overrightarrow{x}\right)=\frac{5{x}_{2}}{{x}_{1}}-1\le 0$$

$${g}_{7}\left(\overrightarrow{x}\right)=\frac{{x}_{2}{x}_{3}}{40}-1\le 0$$

$${g}_{10}\left(\overrightarrow{x}\right)=\frac{1.5{x}_{6}+1.9}{{x}_{4}}-1\le 0$$

$${g}_{9}\left(\overrightarrow{x}\right)=\frac{{x}_{1}}{12{x}_{2}}-1\le 0$$

$${g}_{11}\left(\overrightarrow{x}\right)=\frac{1.1{x}_{7}+1.9}{{x}_{5}}-1\le 0$$

Decision parameter bounds:

$$2.6\le {x}_{1}\le 3.6; 0.7\le {x}_{2}\le 0.8; 17\le {x}_{3}\le 28; 7.3\le {x}_{4}\le 8.3; 7.8\le {x}_{5}\le 8.3; 2.9\le {x}_{6}\le 3.9; 5.0\le {x}_{7}\le 5.5$$

**Figure 17:**
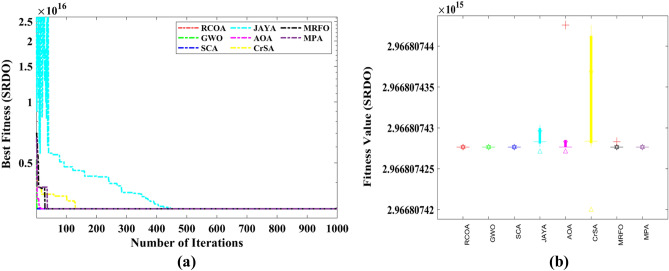
SRDO problem; (**a**) Convergence curves, (**b**) Boxplots.

A total of 11 constraints are regarded in this design problem, seven are nonlinear, and four are linear inequality constraints. The constraints are considered based on (i) stresses in the shafts, (ii) transverse deflections of the shafts, (iii) surface stress, and (iv) bending stress of the gear teeth. Table 6 compares the best optimum solution obtained by using various optimization strategies to compute the best solution. The RCOA requires a total of 1000 iterations to discover a solution. Table 7 compares the statistical findings of RCOA with those of seven optimization strategies and shows which method is superior. The RCOA has produced optimal results when contrasted to the other optimizers, and the findings are superior to those obtained by the other approaches. Figure 17 illustrates the boxplot analysis and convergence curves of all algorithms. The convergence curves and the boxplots conclude that the suggested RCOA can produce the best results with a high convergence speed and better reliability. From the FRT values, it is observed that the RCOA stood first among all chosen algorithms. The RT values represent the computation time required to solve the SRDO problem.

**Table 6:** Decision parameters obtained by selected algorithms for the SRDO challenge.

Method	${x}_{1}$	${x}_{2}$	${x}_{3}$	${x}_{4}$	${x}_{5}$	${x}_{6}$	${x}_{7}$
RCOA	3.600	0.800	17.000	7.300	7.800	3.900	5.500
GWO	3.600	0.800	17.000	7.300	7.800	3.900	5.500
SCA	3.600	0.800	17.000	7.300	7.800	3.900	5.500
JAYA	3.022	0.800	17.000	7.300	7.800	3.900	5.500
AOA	3.600	0.800	17.000	7.300	7.800	3.900	5.500
CrSA	3.600	0.800	17.000	7.300	7.800	3.900	5.500
MRFO	3.600	0.800	17.000	7.300	7.800	3.900	5.500
MPA	3.600	0.800	17.000	7.300	7.800	3.900	5.500

**Table 7:** Statistical analysis of all algorithms on the SRDO problem.

Algorithm	Min	Mean	STD	RT	FRT
RCOA	**2.967E + 15**	**2.967E + 15**	**0.000E + 00**	0.080729	**3.000**
GWO	**2.967E + 15**	**2.967E + 15**	**0.000E + 00**	0.109375	**3.000**
SCA	**2.967E + 15**	**2.967E + 15**	**0.000E + 00**	0.096354	**3.000**
JAYA	**2.967E + 15**	**2.967E + 15**	1.118E + 06	**0.002604**	7.000
AOA	**2.967E + 15**	**2.967E + 15**	6.038E + 06	0.096354	5.833
CrSA	**2.967E + 15**	**2.967E + 15**	7.142E + 06	0.447917	6.417
MRFO	**2.967E + 15**	**2.967E + 15**	2.659E + 05	11.55729	4.750
MPA	**2.967E + 15**	**2.967E + 15**	**0.000E + 00**	1.401054	**3.000**

Significant values are in bold.

#### Three-bar truss design optimization (TTDO) problem

**Figure 18:**
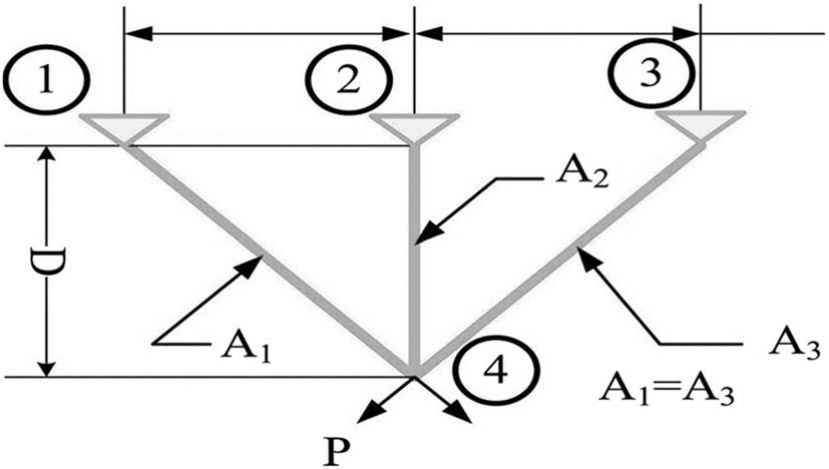
Structure of 3-bar truss.

Figure 17 depicts an illustration of the three-bar truss design in operation. It comprises real-valued and deflection, non-linear buckling, stress limitations, and several other constraints. This system aims to determine the ideal quantities of cross-sectional areas in a given circumstance. Furthermore, the mathematical representations of the model parameters in the fitness function are indicated by ${x}_{1}$ and ${x}_{2}$. Furthermore, in Fig. 18, ‘$P$’ represents the load applied to the truss and ‘$D$’ represents the symmetrical length of the truss.

30 $$\mathrm{Min}: f\left(\overline{x }\right)=D\left({x}_{2}+2\sqrt{2}{x}_{1}\right),$$

Subjected to the following constraints,

$${g}_{1}\left(\overline{x }\right)=\frac{{x}_{2}}{2{x}_{2}{x}_{1}+\sqrt{2}{x}_{1}^{2}}P-\sigma \le 0$$

$${g}_{2}\left(\overline{x }\right)=\frac{{x}_{2}+\sqrt{2}{x}_{1}}{2{x}_{2}{x}_{1}+\sqrt{2}{x}_{1}^{2}}P-\sigma \le 0$$

$${g}_{3}\left(\overline{x }\right)=\frac{1}{{x}_{1}+\sqrt{2}{x}_{2}}P-\sigma \le 0$$

where $$D=100,\,P=2kN/{cm}^{2}, \mathrm{and\, }\sigma =2\;kN/{cm}^{2}$$.

Decision parameter bounds:

$$0\le {x}_{1},{x}_{2}\le 1$$

**Figure 19:**
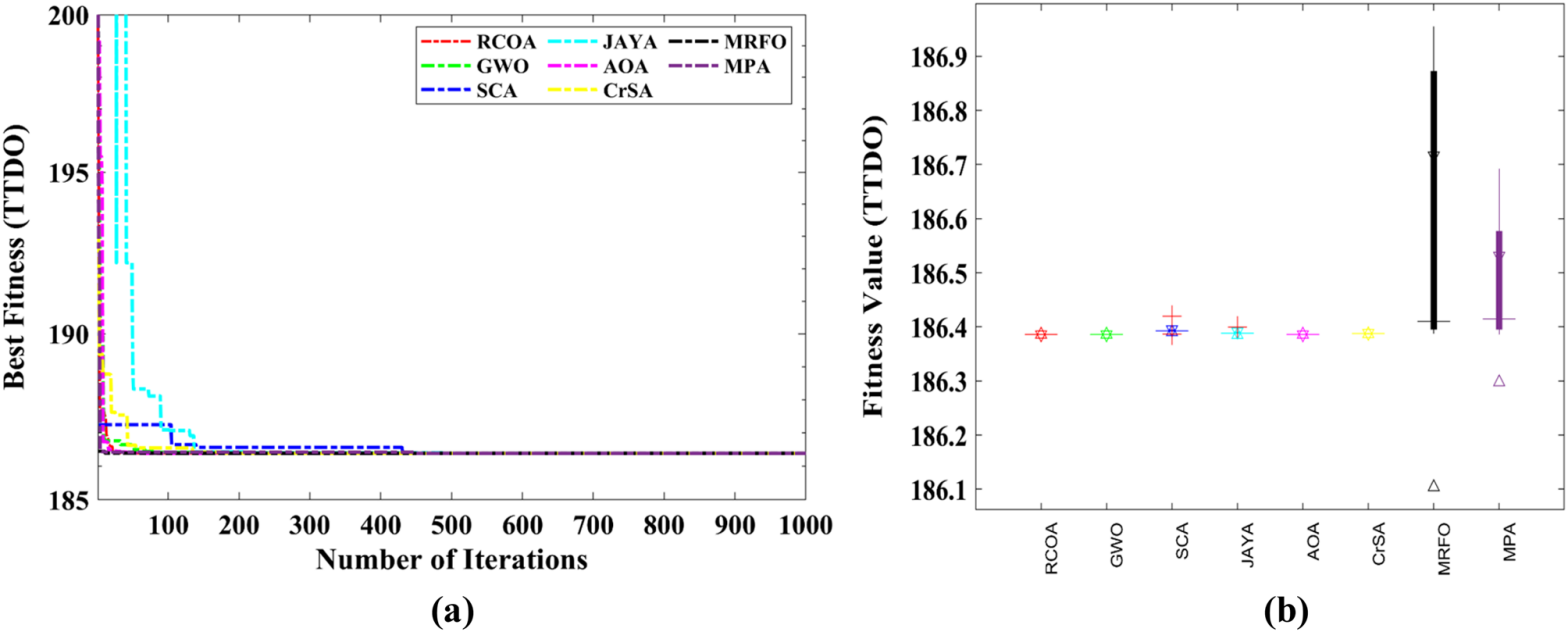
TTDO problem; (**a**) Convergence curves, (**b**) Boxplots.

Table 8 shows the outcomes of the several strategies that produced the best results. Furthermore, the summary statistics of such algorithms are also presented in Table 8. It can be shown that the optimum fitness value of the RCOA is on par with or better than the optimum fitness value of the other approaches. Figure 19 illustrates the convergence graphs and boxplot analysis of all algorithms. The convergence graphs and the boxplot conclude that the RCOA can produce the best results with a high convergence speed and better reliability. From the FRT values, it is observed that the RCOA stood first among all chosen algorithms. The RT values represent the computation time required to solve the TTDO problem.

**Table 8:** Decision vectors obtained by chosen algorithms for the TTDO problem.

Algorithm	${x}_{1}$	${x}_{2}$	Min	Mean	STD	RT	FRT
RCOA	0.7868	0.2880	**186.386**	**186.386**	**2.542E−14**	0.049479	**1.500**
GWO	0.7869	0.2878	**186.386**	**186.386**	1.422E−05	0.049479	3.000
SCA	0.7876	0.2862	186.387	186.396	1.164E−02	0.049479	6.167
JAYA	0.7868	0.2880	**186.386**	186.390	5.117E−03	**0.021421**	5.167
AOA	0.7868	0.2880	**186.386**	**186.386**	**2.542E−14**	0.057292	**1.500**
CrSA	0.7862	0.2893	**186.386**	186.387	8.731E−04	0.388021	4.667
MRFO	0.7885	0.2847	186.387	186.572	2.666E−01	11.59635	7.167
MPA	0.7865	0.2887	**186.386**	186.480	1.259E−01	1.1875	6.833

Significant values are in bold.

#### Pressure vessel design optimization (PVDO) problem

**Figure 20:**
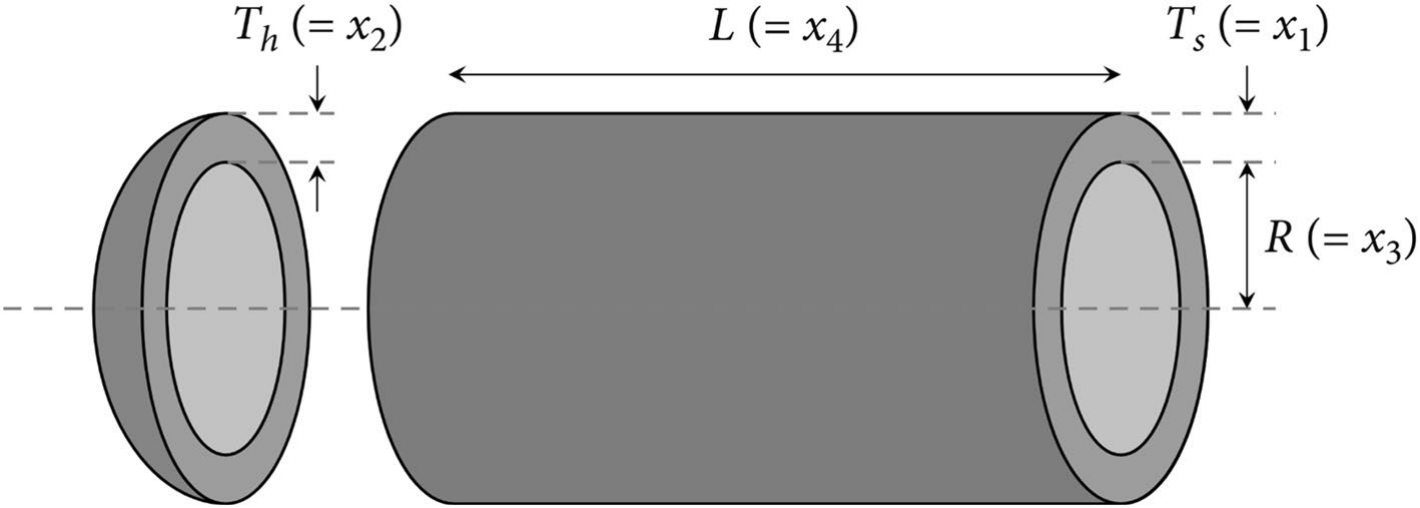
Structure of pressure vessel.

This example is illustrated in Fig. 20, which shows a cylindrical vessel with hemispherical heads on both sides. Overall cost minimization is desired, including material costs and welding and forming costs. In this equation, there are four parameters, including the length of the cylindrical section of the vessel, excluding the head $L$ (${{\varvec{x}}}_{4}$), the thickness of the head ${T}_{h}$ (${{\varvec{x}}}_{3}$), the thickness of the shell ${T}_{s}$ (${{\varvec{x}}}_{2}$), and the radius of the inner cylinder $R$ (${{\varvec{x}}}_{1}$). Furthermore, the variables ${{\varvec{x}}}_{1}$ and ${{\varvec{x}}}_{2}$ are integral multiples of 0.0625 inches, whereas the other parameters are continuous multiples of 0.0625 inches. Equation (31) describes the objective function of the PVDO problem.

31 $$\text{Min: }f\left(x\right)=0.6224{x}_{1}{x}_{3}{x}_{4}+1.7781{x}_{2}{x}_{3}^{2}+3.1661{x}_{1}^{2}{x}_{4}+19.84{x}_{1}^{2}{x}_{3},$$

Subjected to:

$${g}_{2}\left(x\right)=-{x}_{2}+0.00954{x}_{3}\le 0$$

$${g}_{1}\left(x\right)=-{x}_{1}+0.0193{x}_{3}\le 0$$

$${g}_{4}\left(x\right)={x}_{4}-240\le 0$$

$${g}_{3}\left(x\right)=-\pi {x}_{3}^{2}{x}_{4}-\left(4/3\right)\pi {x}_{3}^{3}+1296000\le 0$$

Decision parameter bounds:

$$10\le {x}_{4},{x}_{3}\le 200$$; $$1\le {x}_{2},{x}_{1}\le 99\, (\text{integer\, variables})$$

**Figure 21:**
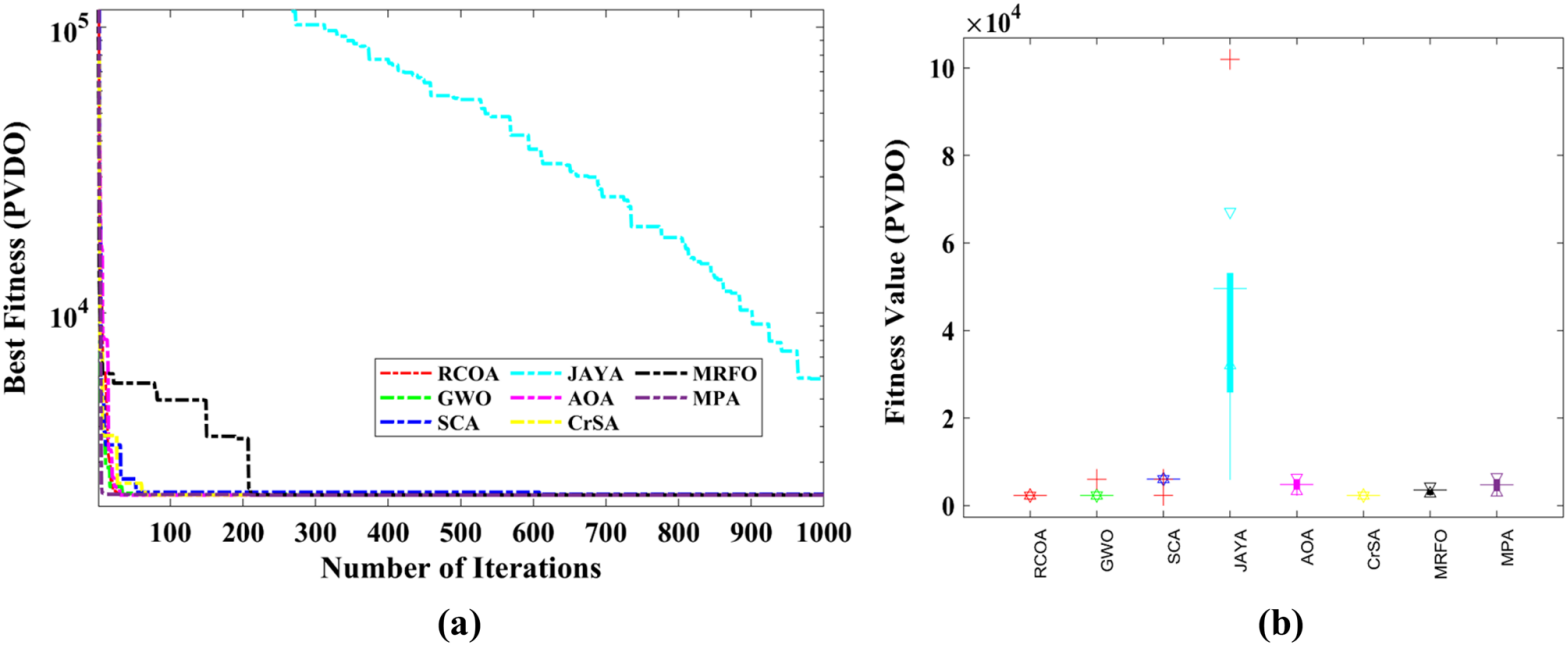
PVDO problem; (**a**) Convergence curves, (**b**) Boxplots.

Table 9 shows the outcomes of the several strategies that produced the best results. Furthermore, the summary statistics of such algorithms are also presented in Table 10. It can be shown that the optimum fitness value of the RCOA is on par with or better than the optimum fitness value of the other algorithms. Figure 21 illustrates the convergence graphs and boxplot analysis of all algorithms. The convergence curves and the boxplot conclude that the suggested RCOA can produce the best results with a high convergence speed and better reliability. From the FRT values, it is observed that the RCOA raised first among all chosen methods. The RT values represent the computation time required to solve the PVDO problem.

**Table 9:** Decision parameters obtained by all selected algorithms for the PVDO problem.

Algorithm	${x}_{1}$	${x}_{2}$	${x}_{3}$	${x}_{4}$
RCOA	1.0936	0.0000	65.2252	10.0000
GWO	1.0893	0.0000	65.2246	10.0036
SCA	1.0991	0.0000	65.3729	10.0000
JAYA	1.1016	0.4501	61.2252	29.2677
AOA	1.0936	0.0000	65.2252	10.0000
CrSA	1.0850	0.0000	65.2273	10.0000
MRFO	1.0771	0.0000	65.2252	10.0001
MPA	1.0906	0.0000	65.2252	10.0000

**Table 10:** Statistical analysis of the chosen methods for the PVDO problem.

Method	Min	Mean	STD	RT	FRT
RCOA	**2302.546**	**2302.546**	**4.982E−13**	0.055	**1.000**
GWO	2302.861	2938.773	1.527E + 03	0.063	3.333
SCA	2316.624	5434.068	1.527E + 03	0.060	6.500
JAYA	5879.312	47,666.365	3.229E + 04	**0.003**	8.000
AOA	**2302.546**	4619.586	1.645E + 03	0.065	4.500
CrSA	2303.581	2305.337	1.415E + 00	0.398	2.667
MRFO	2305.618	3188.695	6.581E + 02	11.497	4.833
MPA	2302.644	4548.721	1.709E + 03	1.021	5.167

Significant values are in bold.

#### Tubular column design optimization (TCDO) problem

**Figure 22:**
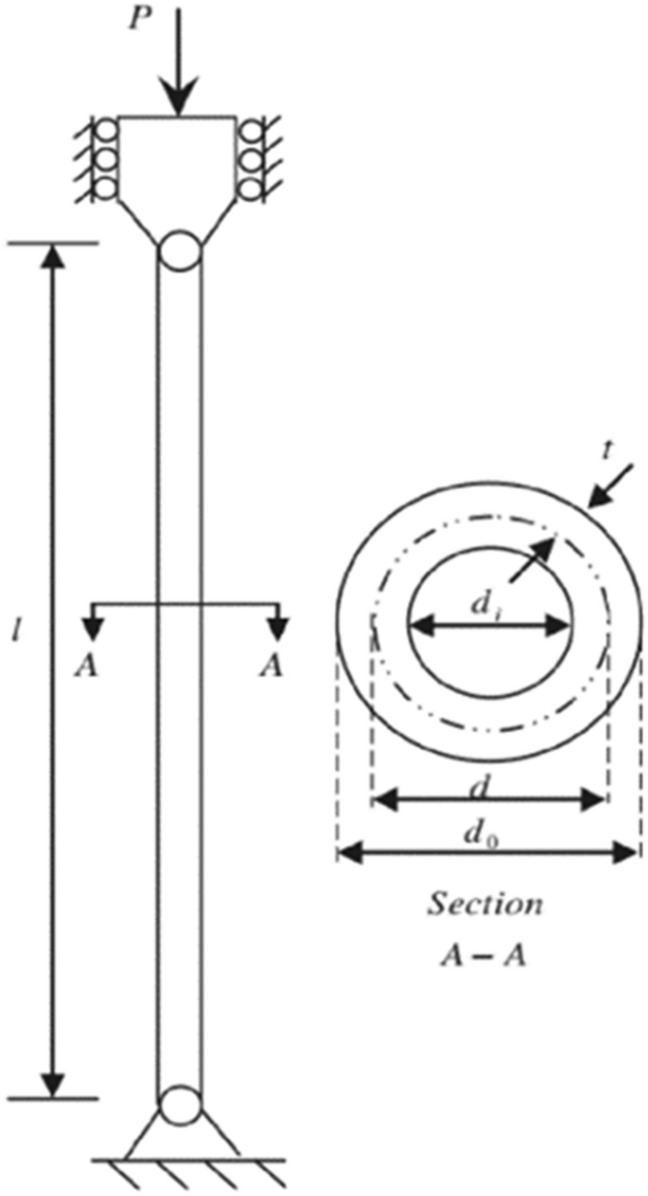
Structure of the tubular column.

This challenge aims to find the most cost-effective materials and building methods for a tubular column. There are two decision parameters, including the mean column section thickness (${x}_{1}$) and mean column section diameter (${x}_{2}$), as well as six inequality constraints. It is illustrated in Fig. 22 how this problem is presented schematically.

The fitness function and constraints of the TCDO are provided below.

32 $${\text{Min}}:f\left(x\right)=9.8{x}_{1}{x}_{2}+2{x}_{1},$$

Subjected to:

$${g}_{1}(x)=\frac{P}{\pi {x}_{1}{x}_{2}{\sigma }_{y}}-1\le 0$$

$${g}_{2}(x)=\frac{8P{L}^{2}}{{\pi }^{3}E{x}_{1}{x}_{2}\left({{x}_{1}}^{2}+{{x}_{2}}^{2}\right)}-1\le 0$$

$${g}_{3}(x)=\frac{2.0}{{x}_{1}}-1\le 0$$

$${g}_{4}(x)=\frac{{x}_{1}}{14}-1\le 0$$

$${g}_{5}(x)=\frac{0.2}{{x}_{2}}-1\le 0$$

$${g}_{6}(x)=\frac{{x}_{2}}{0.8}-1\le 0$$

where $${\sigma }_{y}=500 \mathrm{kgf}/{\mathrm{cm}}^{2}$$, $$\mathrm{P}=2500 \mathrm{kgf}$$, $$L=250 \mathrm{cm}$$ and $$E=0.85\times {10}^{6}\mathrm{ kgf}/{\mathrm{cm}}^{2}$$.

Decision parameter bounds:

$$0.2\le {x}_{2}\le 0.8$$; $$2\le {x}_{1}\le 14.$$

**Figure 23:**
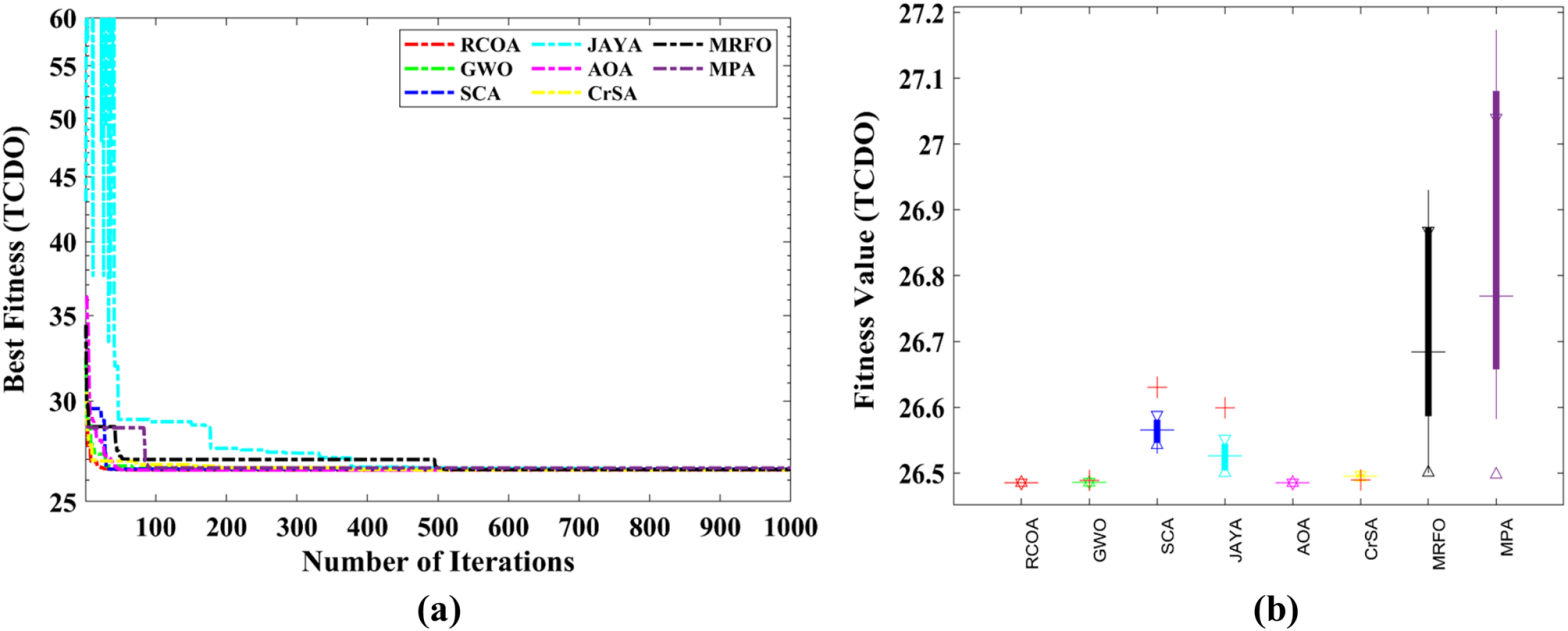
TCDO problem; (**a**) Convergence curves, (**b**) Boxplots.

Table 11 shows the outcomes of the several strategies that produced the best results. Furthermore, the summary statistics of such algorithms are also presented in Table 11. It can be shown that the optimum fitness value of the RCOA is on par with or higher than the optimum fitness value of the other algorithms. Figure 23 illustrates the convergence graphs and boxplot analysis of all algorithms. The convergence curves and the boxplot conclude that the suggested RCOA can produce the best results with a high convergence rate and better reliability. From the FRT values, it is observed that the RCOA raised first among all chosen methods. The RT values represent the computation time required to solve the TTDO problem.

**Table 11:** Decision parameters obtained by all algorithms for the TCDO problem.

Algorithm	${x}_{1}$	${x}_{2}$	Min	Mean	STD	RT	FRT
RCOA	5.4527	0.2915	**26.4854**	**26.4854**	**3.892E−15**	0.049342	**1.000**
GWO	5.4528	0.2915	26.4855	26.4865	1.146E−03	0.052083	3.000
SCA	5.4631	0.2914	26.5297	26.5698	3.477E−02	0.049479	6.167
JAYA	5.4599	0.2912	26.5032	26.5341	3.648E−02	**0.002604**	5.333
AOA	5.4527	0.2915	**26.4854**	**26.4854**	1.989E−09	0.054688	2.000
CrSA	5.4542	0.2915	26.4896	26.4954	3.390E−03	0.390625	4.000
MRFO	5.4484	0.2922	26.5002	26.7097	1.654E−01	11.54167	7.000
MPA	5.5014	0.2889	26.5827	26.8388	2.422E−01	1.184896	7.500

Significant values are in bold.

#### Corrugated bulkhead design optimization (CBDO) problem

When designing a corrugated bulkhead for a chemical container, it is important to consider the width (${{\varvec{x}}}_{1}$), the depth (${{\varvec{x}}}_{2}$), the length (${{\varvec{x}}}_{3}$), and the thickness of the plate (${{\varvec{x}}}_{4}$). As an example of a mathematical formula for this optimization problem, consider the following:

33 $${\text{Min}}:f\left(x\right)=\frac{5.885{x}_{2}\left({x}_{1}+{x}_{3}\right)}{{x}_{1}+\sqrt{\left({{x}_{3}}^{2}-{{x}_{2}}^{2}\right)}},$$

Subjected to:

$${g}_{1}(x)={x}_{4}{x}_{2}\left(0.4{x}_{1}+\frac{{x}_{3}}{6}\right)-8.94\left({x}_{1}+\sqrt{\left({{x}_{3}}^{2}-{{x}_{2}}^{2}\right)}\right)\ge 0$$

$${g}_{2}(x)={x}_{4}{{x}_{2}}^{2}\left(0.2{x}_{1}+\frac{{x}_{3}}{12}\right)-2.2{\left(8.94\left({x}_{1}+\sqrt{\left({{x}_{3}}^{2}-{{x}_{2}}^{2}\right)}\right)\right)}^\frac{4}{3}\ge 0$$

$${g}_{3}(x)={x}_{4}-0.0156{x}_{1}-0.15\ge 0$$

$${g}_{4}(x)={x}_{4}-0.0156{x}_{3}-0.15\ge 0$$

$${g}_{5}(x)={x}_{4}-1.05\ge 0$$

$${g}_{6}(x)={x}_{3}-{x}_{2}\ge 0$$

Decision parameter bounds:

$$0\le {x}_{1},{x}_{2},{x}_{3}\le 100$$; $$0\le {x}_{4}\le 5.$$

**Figure 24:**
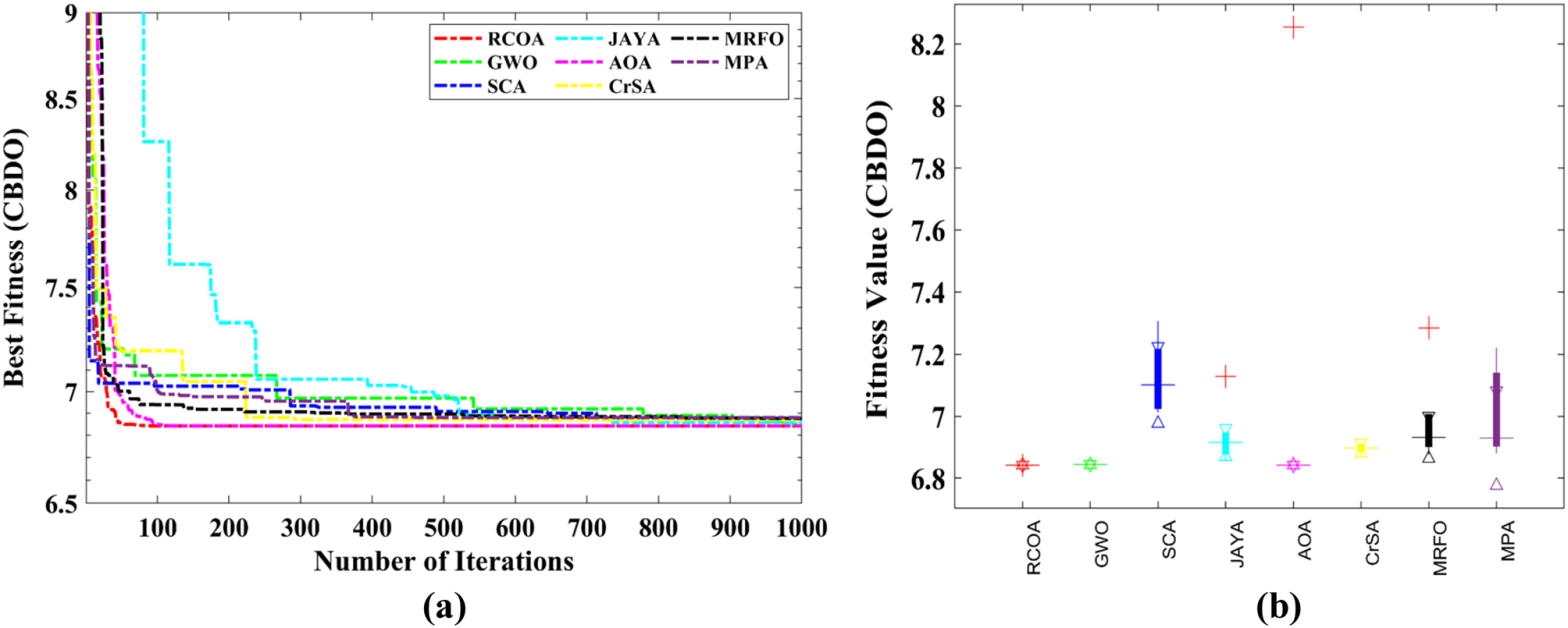
TTDO problem; (**a**) Convergence curves, (**b**) Boxplots.

Table 12 shows the outcomes of the several strategies that produced the best results. Furthermore, the summary statistics of such algorithms are also presented in Table 13. It can be shown that the optimum fitness value of the RCOA is on par with or higher than the optimum fitness value of the other methods. Figure 24 illustrates the convergence graphs and boxplot analysis of all algorithms. The convergence graphs and the boxplot conclude that the RCOA can produce the best results with a high convergence speed and better reliability. From the FRT values, it is observed that the RCOA stood first among all chosen algorithms. The RT values represent the computation time required to solve the TTDO problem.

**Table 12:** Decision vectors found by chosen methods for the TTDO problem.

Algorithm	${x}_{1}$	${x}_{2}$	${x}_{3}$	${x}_{4}$
RCOA	57.6790	34.1489	57.6808	1.0498
GWO	57.5824	34.1486	57.6491	1.0497
SCA	54.4043	34.1727	57.6663	1.0516
JAYA	57.0414	34.3216	57.6047	1.0502
AOA	57.6791	34.1489	57.6808	1.0498
CrSA	56.3816	34.2919	57.2565	1.0491
MRFO	57.2006	33.9474	56.6920	1.0526
MPA	56.6587	34.1091	58.0464	1.0562

**Table 13:** Statistical analysis of all methods on the TTDO challenge.

Algorithm	Min	Mean	STD	RT	FRT
RCOA	**6.8424**	**6.8424**	**1.299E−10**	0.059896	**1.167**
GWO	6.8436	6.8441	4.736E−04	0.072917	3.000
SCA	6.8769	6.8836	1.119E−02	0.0625	6.833
JAYA	6.8589	6.9404	9.845E−02	**0.03564**	5.167
AOA	**6.8424**	7.0778	5.766E−01	0.070313	3.000
CrSA	6.8704	6.8955	1.788E−02	0.445313	4.667
MRFO	6.8761	6.9887	1.514E−01	12.48438	6.000
MPA	6.8802	7.0004	1.432E−01	1.09375	6.167

Significant values are in bold.

#### Tension/compression spring design optimization (TCSDO) problem

**Figure 25:**
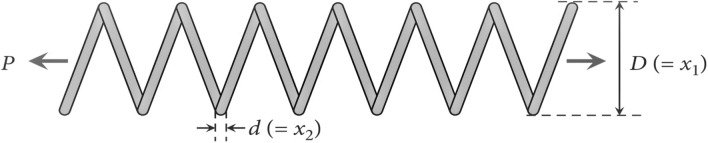
Schematic of tension/compression spring.

The Tension/Compression Spring Design Optimization (TCSDO) problem has been designed as an alternative to the typical mechanical design problem of flexing springs. In Fig. 25, we can see that the core objective is to reduce the tension spring-mass of the structure to the absolute minimum. $x=\left[{x}_{1},{x}_{2},{x}_{3}\right]=$[$D,d,N$] is considered when solving the TCSDO problem. The active coils ($N$), wire diameter ($d$), and mean coil diameter ($D$) are considered in the solution.

The objective function and constraints of the TCSDO are presented below.

34 $$\text{Min: }f\left(\overrightarrow{x}\right)=\left({x}_{3}+2\right){x}_{2}{x}_{1}^{2},$$

Subjected to:

$${g}_{1}\left(\overrightarrow{x}\right)=1-\frac{{x}_{2}^{3}{x}_{3}}{71785{x}_{1}^{4}}\le 0$$

$${g}_{2}\left(\overrightarrow{x}\right)=\frac{4{x}_{2}^{2}-{x}_{1}{x}_{2}}{12566\left({x}_{2}{x}_{1}^{3}-{x}_{1}^{4}\right)}+\frac{1}{5108{x}_{1}^{2}}-1\le 0$$

$${g}_{3}\left(\overrightarrow{x}\right)=1-\frac{140.45{x}_{1}}{{x}_{2}^{2}{x}_{3}}\le 0$$

$${g}_{4}\left(\overrightarrow{x}\right)=\frac{{x}_{1}+{x}_{2}}{1.5}-1\le 0$$

Decision parameter bounds:

$$0.05\le {x}_{1}\le 2.0, 0.25\le {x}_{2}\le 1.3,\text{ and }2\le {x}_{3}\le 15.0.$$

**Figure 26:**
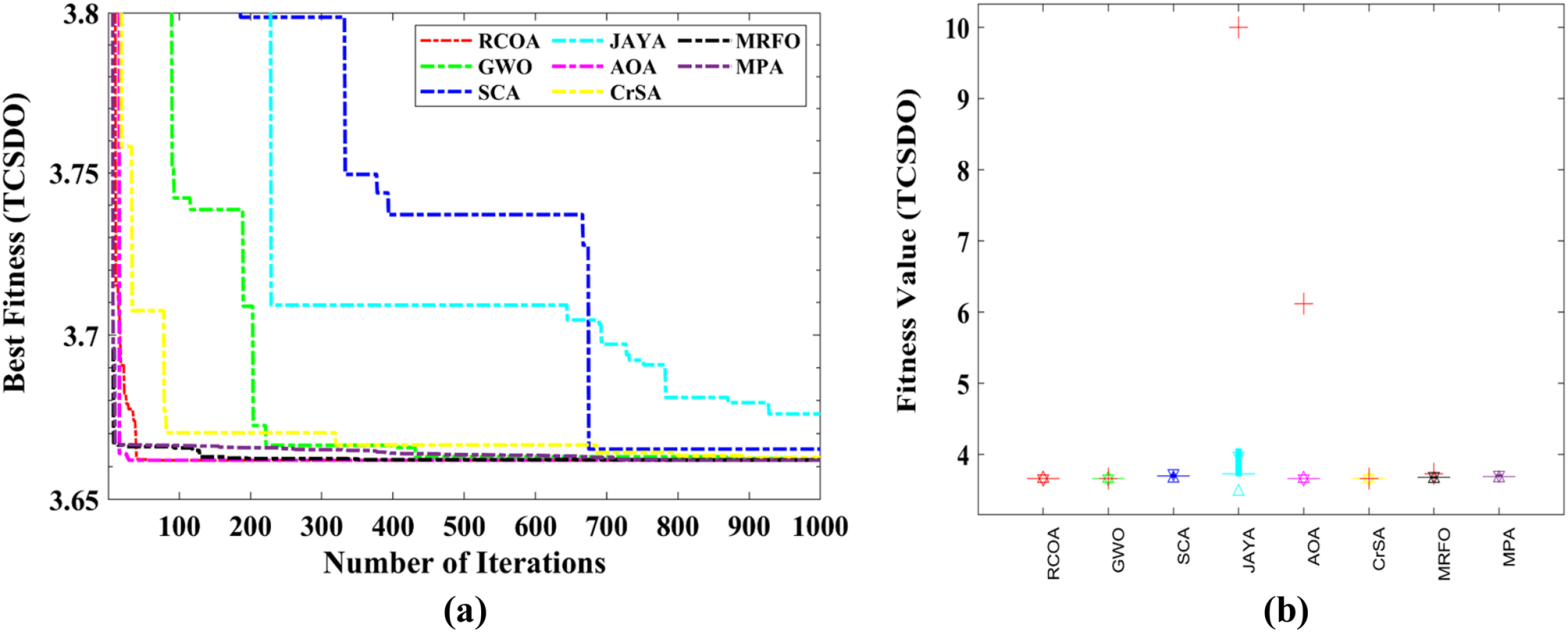
TCSDO problem; (**a**) Convergence curves, (**b**) Boxplots.

Table 14 shows the outcomes of the several strategies that produced the best results. Furthermore, the summary statistics of such algorithms are also presented in Table 15. It can be shown that the optimum fitness value of the RCOA is on par with or higher than the optimum fitness value of the other algorithms. Figure 26 illustrates the convergence graphs and boxplot analysis of all algorithms. The convergence graphs and the boxplot conclude that the RCOA can produce the best results with a high convergence speed and better reliability. From the FRT values, it is observed that the RCOA stood first among all chosen algorithms. The RT values represent the computation time required to solve the TCSDO problem.

**Table 14:** Decision parameters found by all methods for the TCSDO challenge.

Algorithm	${x}_{1}$	${x}_{2}$	${x}_{3}$
RCOA	0.1391	1.3000	11.8924
GWO	0.1392	1.3000	11.8915
SCA	0.1388	1.3000	11.8200
JAYA	0.1381	1.2672	12.4117
AOA	0.1391	1.3000	11.8924
CrSA	0.1391	1.2987	11.9305
MRFO	0.1392	1.3000	11.9019
MPA	0.1391	1.3000	11.8909

**Table 15:** Statistical analysis of the selected methods on the TCSDO challenge.

Method	Min	Mean	STD	RT	FRT
RCOA	**3.6619**	**3.6619**	**3.972E−16**	0.0729	**1.000**
GWO	**3.6619**	**3.6619**	7.647E−06	0.0911	3.000
SCA	3.6653	3.7033	3.386E−02	0.0781	6.500
JAYA	3.6760	4.8195	2.543E + 00	**0.0052**	7.333
AOA	**3.6619**	4.0710	1.002E + 00	0.0877	3.000
CrSA	3.6627	3.6632	7.107E−04	0.4141	4.167
MRFO	3.6620	3.6849	2.608E−02	11.9010	5.167
MPA	**3.6619**	3.6975	2.570E−02	1.3411	5.833

Significant values are in bold.

#### Welded beam design optimization (WBDO) problem

**Figure 27:**
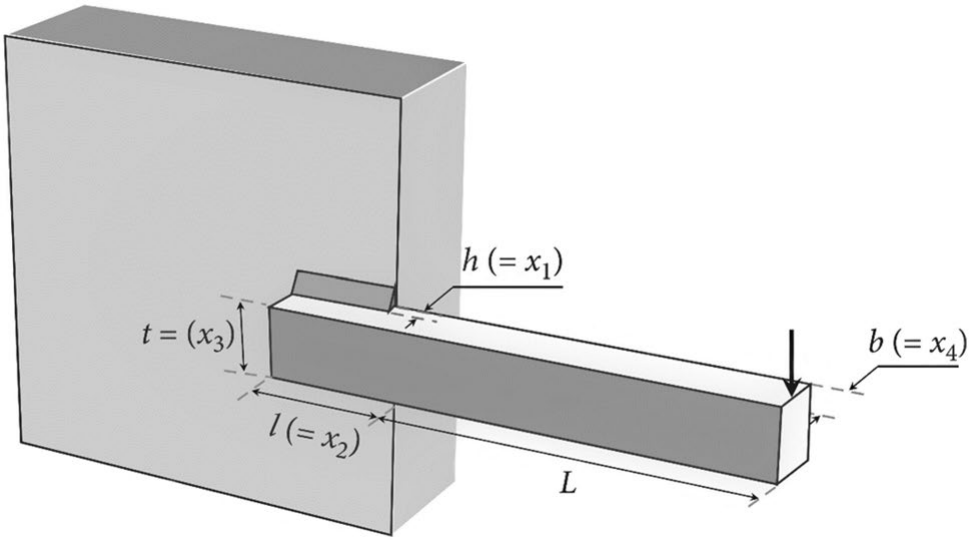
Schematic of a welded beam.

This WBDO is a standard test problem, and it has since been solved by a large group of experts. The beam is subjected to a vertical force, as shown in Fig. 27. With this problem, the objective is to discover the welded beam with the lowest cost of manufacture. Geometry, welding, deflection, and stress are the seven constraints the problem must address. The decision parameters are as follows: weld thickness (${{\varvec{x}}}_{1}$), height (${{\varvec{x}}}_{2}$), length (${{\varvec{x}}}_{3}$), and bar thickness (${{\varvec{x}}}_{4}$), as depicted in Fig. 27. Using mathematics, the objective function may be expressed as follows.

35 $$\mathrm{Min}: f\left(x\right)=1.10471{x}_{1}^{2}{x}_{2}+0.04811\left(14+{x}_{2}\right){x}_{3}{x}_{4},$$

Subjected to:

$${g}_{1}\left(x\right)=\tau \left(\mathrm{x}\right)-{\tau }_{max}\le 0$$

$${g}_{2}\left(x\right)=\sigma \left(x\right)-{\sigma }_{max}\le 0$$

$${g}_{3}\left(x\right)=\delta \left(x\right)-{\delta }_{max}\le 0$$

$${g}_{4}\left(x\right)={x}_{1}-{x}_{4}\le 0$$

$${g}_{5}\left(x\right)=P-{P}_{c}\left(x\right)\le 0$$

$${g}_{6}\left(x\right)=0.125-{x}_{1}\le 0$$

$${g}_{7}\left(x\right)=1.10471{x}_{1}^{2}+0.04811{x}_{3}{x}_{4}\left(14.0+{x}_{2}\right)-5.0\le 0$$

where $$P=6000 lb,L=14 inch,{\delta }_{max}=0.25 \; inch, E=30\times {1}^{6}psi,G=12\times {10}^{6}psi,$$ and $${\tau }_{max}=13600 psi,{\sigma }_{max}=30000 psi$$.

Decision parameter bounds:

$$0.1\le {x}_{1}\le 2$$, $$0.1\le {x}_{2}\le 10$$, $$0.1\le {x}_{3}\le 10$$, and $$0.1\le {x}_{4}\le 2$$.

**Figure 28:**
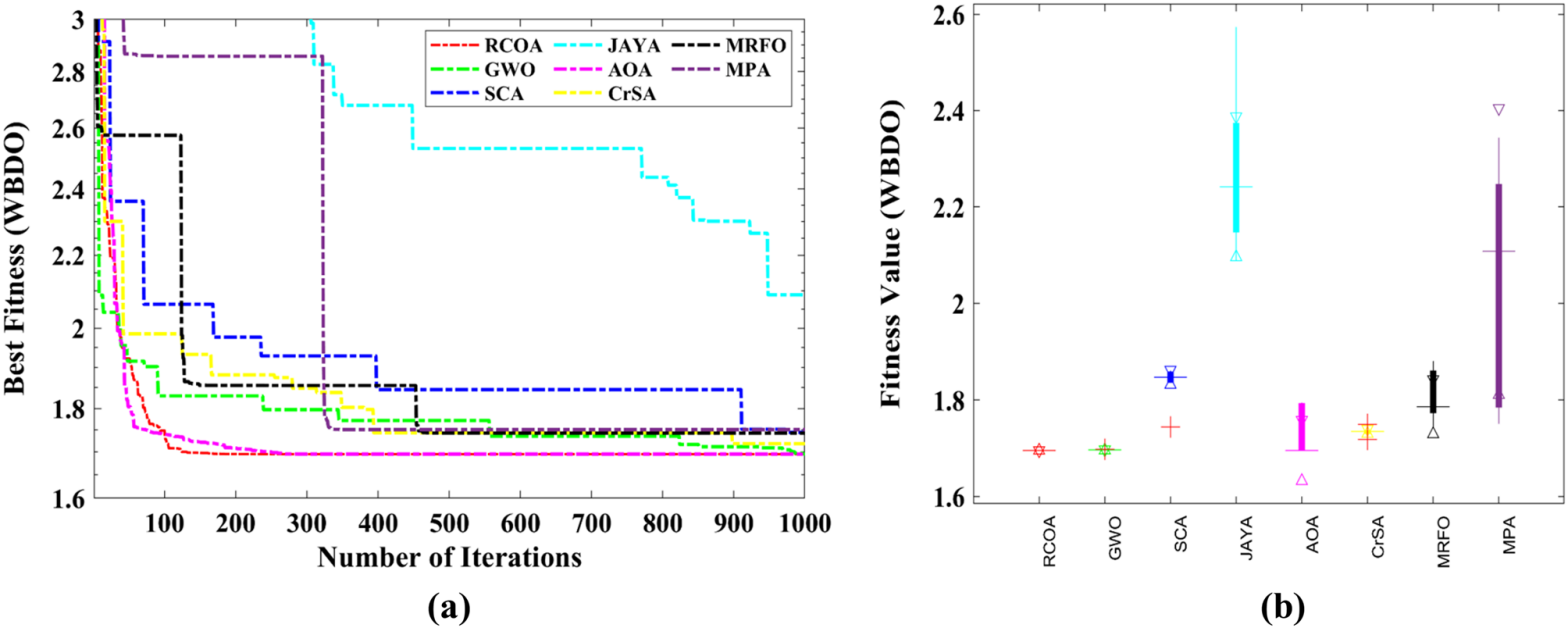
WBDO problem; (**a**) Convergence curves, (**b**) Boxplots.

Table 16 shows the outcomes of the several strategies that produced the best results. Furthermore, the summary statistics of such algorithms are also presented in Table 17. It can be shown that the optimum fitness value of the RCOA is on par with or better than the optimum fitness value of the other algorithms. Figure 28 illustrates the convergence graphs and boxplot analysis of all algorithms. The convergence graphs and the boxplot conclude that the RCOA can produce the best results with a high convergence speed and better reliability. From the FRT values, it is observed that the RCOA stood first among all chosen algorithms. The RT values represent the computation time required to solve the WBDO problem.

**Table 16:** Decision parameters found by all methods for the WBDO challenge.

Algorithm	${x}_{1}$	${x}_{2}$	${x}_{3}$	${x}_{4}$
RCOA	0.2057	3.2530	9.0366	0.2057
GWO	0.2055	3.2577	9.0371	0.2058
SCA	0.1875	3.6173	9.1762	0.2062
JAYA	0.2989	2.5867	7.5377	0.3048
AOA	0.2057	3.2530	9.0366	0.2057
CrSA	0.2039	3.3202	9.1054	0.2064
MRFO	0.1993	3.3704	9.0422	0.2111
MPA	0.1814	3.8315	9.1567	0.2051

**Table 17:** Statistical analysis of all methods on the WBDO challenge.

Method	Min	Mean	STD	RT	FRT
RCOA	**1.6952**	**1.6952**	**3.074E−09**	0.1172	**1.000**
GWO	1.6958	1.6967	8.137E−04	0.1667	2.667
SCA	1.7444	1.8325	4.417E−02	0.1276	5.667
JAYA	2.0886	2.2779	1.755E−01	**0.0104**	7.833
AOA	**1.6952**	1.7283	5.114E−02	0.1276	2.667
CrSA	1.7187	1.7342	1.011E−02	0.5781	3.667
MRFO	1.7427	1.8050	5.465E−02	12.3646	5.833
MPA	1.7507	2.0570	2.454E−01	2.5911	6.667

Significant values are in bold.

#### Car side impact design optimization (CSIDO) problem

**Figure 29:**
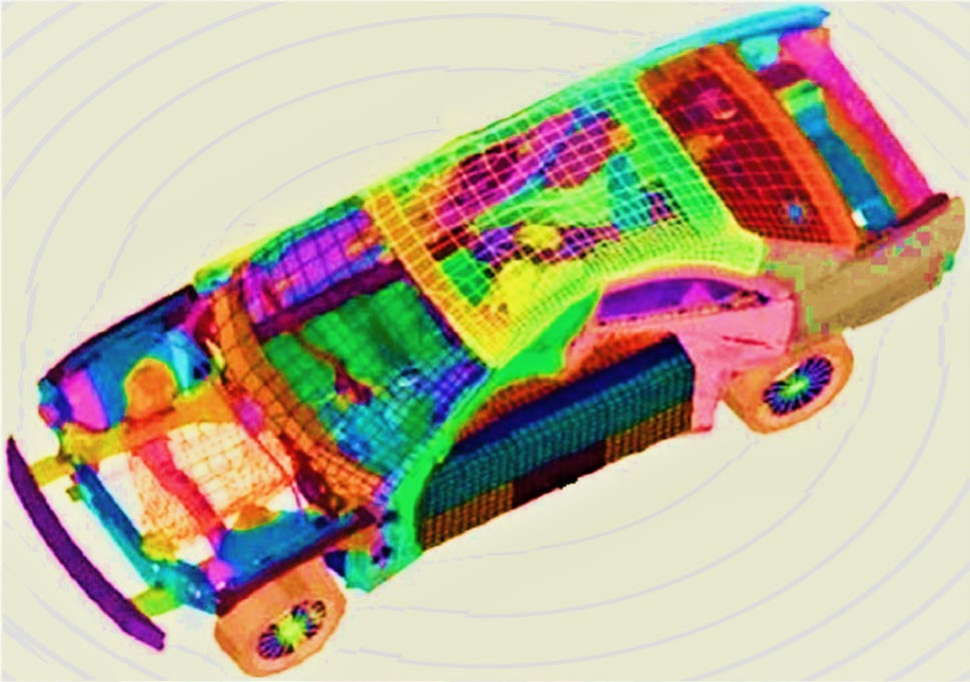
FEM model of car side impact.

The proposed RCOA uses the design of an automobile side impact as a benchmark challenge. Figure 29 depicts a representation of the FEM model for this application. A car is subjected to a side-impact test based on protocols developed by the European enhanced vehicle safety committee. The purpose is to decrease the weight by controlling nine impact parameters, including the thicknesses of the roof rail, beltline reinforcement, door beam, cross members, floor side inner, B-Pillar reinforcement, and B-Pillar inner (${x}_{1}-{x}_{7}$), the materials of B-Pillar inner and floor side inner (${x}_{8}-{x}_{9}$) and the height of the barrier (${x}_{10}-{x}_{11}$).

The fitness function and constraints of the CSIDO are provided below.

36 $$\mathrm{Min}: f\left({\varvec{x}}\right):=1.98+2.73{x}_{7}+4.90{x}_{1}+1.78{x}_{5}+4.01{x}_{4}+6.67{x}_{2}+6.98{x}_{3},$$

Subjected to:

$${g}_{1}(x)={F}_{a}(\text{load in the abdomen})\le 1{\text{kN}}$$

$${g}_{2}\left(x\right)={\mathrm{VC}}_{u}\left(\text{dummy upper chest}\right)\le 0:\frac{32{\text{m}}}{\text{s}}$$

$${g}_{3}\left(x\right)={\mathrm{VC}}_{m}\left(\text{dummy middle chest}\right)\le 0:\frac{32{\text{m}}}{\text{s}}$$

$${g}_{4}\left(x\right)={\mathrm{VC}}_{l}\left(\text{dummy lower chest}\right)\le 0:\frac{32{\text{m}}}{\text{s}}$$

$${g}_{5}\left(x\right)={\Delta }_{\text{ur}}\left(\text{upper rib deflection}\right)\le 32{\text{mm}}$$

$${g}_{7}(x)={\Delta }_{\text{lr}}(\text{lower rib deflection})\le 32{\text{mm}}$$

$${g}_{6}(x)={\Delta }_{\text{mr}}(\text{middle rib deflection}\le 32{\text{mm}}$$

$${g}_{8}\left(x\right)={F}_{p}\left(\text{Pubic force}\right)\le 4{\text{kN}}$$

$${g}_{9}(x)={V}_{\text{MBP}}(\text{Velocity of V-Pillar at the middle point})\le 9:9{\text{mm}}/{\text{ms}}$$

$${g}_{10}(x)={\mathrm{V}}_{\text{FD}}(\text{Velocity of the front door at V-Pillar})\le 15:7{\text{mm}}/{\text{ms}}$$

where

$${V}_{FD}=16.45-0.0556{x}_{9}{x}_{11}-0.489{x}_{3}{x}_{7}+0.0432{x}_{9}{x}_{10}-0.843{x}_{5}{x}_{6}-0.000786{x}_{11}^{2}$$

$${V}_{MBP}=10.58-0.674{x}_{1}{x}_{2}-1.95{x}_{2}{x}_{8}+0.02054{x}_{3}{x}_{10}-0.0198{x}_{4}{x}_{10}+0.028{x}_{6}{x}_{10}$$

$${F}_{p}=4.72-0.5{x}_{4}-0.19{x}_{2}{x}_{3}-0.0122{x}_{4}{x}_{10}+0.009325{x}_{6}{x}_{10}+0.000191{x}_{11}^{2}$$

$${\Delta }_{ur}=28.98+3.818{x}_{3}-4.2{x}_{1}{x}_{2}+0.0207{x}_{5}{x}_{10}+6.63{x}_{6}{x}_{9}-7.7{x}_{7}{x}_{8}+0.32{x}_{9}{x}_{10}$$

$${\Delta }_{mr}=33.86+2.95{x}_{3}+0.1792{x}_{10}-5.057{x}_{1}{x}_{2}-11.0{x}_{2}{x}_{8}-0.0215{x}_{5}{x}_{10}-9.98{x}_{7}{x}_{8}+22.0{x}_{8}{x}_{9}$$

$${\Delta }_{lr}=46.36-9.9{x}_{2}-12.9{x}_{1}{x}_{8}+0.1107{x}_{3}{x}_{10}$$

$$V{C}_{l}=0.74-0.61{x}_{2}-0.163{x}_{3}{x}_{8}+0.001232{x}_{3}{x}_{10}-0.166{x}_{7}{x}_{9}+0.227{x}_{2}^{2}$$

$$V{C}_{m}=0.214+0.00817{x}_{5}-0.131{x}_{1}{x}_{8}-0.0704{x}_{1}{x}_{9}+0.03099{x}_{2}{x}_{6}-0.018{x}_{2}{x}_{7}+0.00121{x}_{8}{x}_{11}+0.00184{x}_{9}{x}_{10}-0.02{x}_{2}^{2}$$

$$V{C}_{u}=0.261-0.0159{x}_{1}{x}_{2}-0.188{x}_{1}{x}_{8}-0.019{x}_{2}{x}_{7}+0.0144{x}_{3}{x}_{5}+0.0008757{x}_{5}{x}_{10}+0.08045{x}_{6}{x}_{9}+0.00139{x}_{8}{x}_{11}+0.00001575{x}_{10}{x}_{11}$$

$${F}_{a}=1.16+0.01343{x}_{6}{x}_{10}-0.00931{x}_{2}{x}_{10}-0.3717{x}_{2}{x}_{4}-0.484{x}_{3}{x}_{9}$$

Decision parameter bounds:

$$0.5\le {x}_{1},{x}_{2},{x}_{3},{x}_{4},{x}_{5},{x}_{6},{x}_{7}\le 1.5$$; $${x}_{8},{x}_{9}\in \{\mathrm{0.192,0.345}\}$$, and $$-30\le {x}_{10},{x}_{11}\le +30$$.

**Figure 30:**
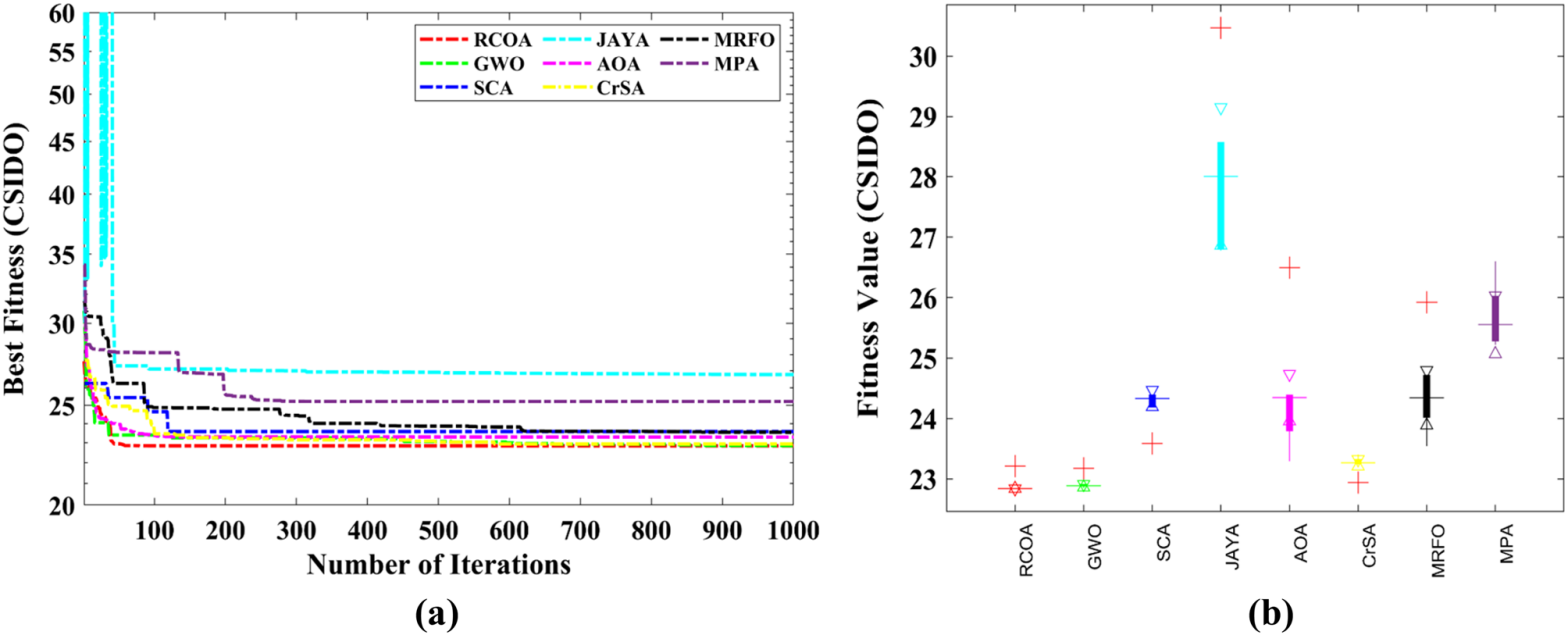
CSIDO problem; (**a**) Convergence curves, (**b**) Boxplots.

Table 18 shows the outcomes of the several strategies that produced the best results. Furthermore, the summary statistics of such algorithms are also presented in Table 19. It can be shown that the optimum fitness value of the RCOA is on par with or higher than the optimum fitness value of the other algorithms. Figure 30 illustrates the convergence graphs and boxplot analysis of all algorithms. The convergence graphs and the boxplot conclude that the suggested RCOA can produce the best results with a high convergence speed and better reliability. From the FRT values, it is observed that the RCOA raised first among all chosen methods. The RT values represent the computation time required to solve the CSIDO problem.

**Table 18:** Decision parameters found by all methods for the CSIDO challenge.

Algorithm	${x}_{1}$	${x}_{2}$	${x}_{3}$	${x}_{4}$	${x}_{5}$	${x}_{6}$	${x}_{7}$	${x}_{8}$	${x}_{9}$	${x}_{10}$	${x}_{11}$
RCOA	0.5000	1.1155	0.5000	1.3007	0.5000	1.5000	0.5000	1.0000	0.7746	− 19.7211	0.0000
GWO	0.5004	1.1072	0.5010	1.3141	0.5000	1.5000	0.5000	0.9298	0.0000	− 21.1258	− 1.0315
SCA	0.5000	1.0424	0.9262	0.8585	0.5000	1.5000	0.5000	0.0000	0.0000	− 30.0000	0.5549
JAYA	0.7145	1.1668	0.7732	1.2139	0.9223	0.9267	0.5892	0.6646	0.6512	4.2538	18.8570
AOA	0.5000	1.2653	0.5000	1.1660	0.5000	0.5000	0.5000	1.0000	1.0000	7.0607	16.3137
CrSA	0.5031	1.1288	0.5006	1.2982	0.5031	1.4980	0.5000	0.9842	0.9264	− 17.6318	4.6333
MRFO	0.5000	1.1046	0.5000	1.4168	0.5470	1.3942	0.5842	0.5000	0.0000	− 21.6960	− 2.4975
MPA	0.5000	1.3753	0.5000	1.0678	1.3924	0.5000	0.5000	0.2894	0.0000	8.9215	10.1099

**Table 19:** Statistical analysis of all methods on the CSIDO problem.

Method	Min	Mean	STD	RT	FRT
RCOA	**22.8370**	**22.8996**	**1.534E−01**	0.067708	**1.167**
GWO	22.8477	22.9288	1.202E−01	0.080729	1.833
SCA	23.5836	24.2038	3.140E−01	0.088542	4.833
JAYA	26.7785	28.1052	1.368E + 00	**0.005208**	8.000
AOA	23.2905	24.4440	1.096E + 00	0.096354	5.333
CrSA	22.9382	23.2399	1.598E−01	0.533854	3.000
MRFO	23.5439	24.4805	8.179E−01	11.61198	5.167
MPA	25.2197	25.7042	5.364E−01	1.354167	6.667

Significant values are in bold.

### Further discussions

The superior performance of the RCOA for numerical and constrained optimization problems can be attributed to several unique characteristics of the algorithm. (i) RCOA is inspired by the time response of the resistance–capacitance circuit, and this inspiration allows it to adeptly handle sudden changes and adapt quickly, enhancing its effectiveness in numerical optimization problems, (ii) Unlike many optimization algorithms that require meticulous tuning of control parameters, RCOA operates devoid of any such parameters. This simplifies the optimization process and increases the algorithm's robustness, thus making it suitable for handling optimization problems, including constrained ones, (iii) The RCOA has a well-defined and balanced exploration and exploitation process. The exploration phase allows the algorithm to search a broad solution space, thus reducing the likelihood of premature convergence to local optima, while the exploitation phase ensures it can effectively navigate towards optimal solutions. This balance makes it particularly adept at solving complex numerical and constrained optimization problems, (iv) As demonstrated in the study, RCOA converges significantly quicker than other algorithms. Fast convergence is particularly crucial in constrained optimization problems, where the solution space is often bounded, and reaching the optimal solution efficiently is of utmost importance, and (v) Unlike some algorithms that perform well on specific functions but poorly on others, RCOA has demonstrated consistent performance across a variety of benchmark functions.

According to the findings, the outcomes of the eight engineering design optimization problems and 23 numerical optimization problems showed that the proposed RCOA was the most effective of all of them. The detailed investigation reveals that the RCOA is superior to other metaheuristic algorithms in terms of performance and efficiency. Based on the outcomes obtained when addressing traditional engineering design problems, it can be inferred that the RCOA outperforms other methods when dealing with all the selected problems compared to other approaches. The suggested RCOA can potentially identify the optimal global solutions to various optimization problems more efficiently than certain algorithms currently available in the scientific literature. Because of RCOA's exploratory and exploitation capabilities, it can produce and enhance a set of potential solutions for an optimization problem in a stochastically generated way. Furthermore, the RCOA has no tunable parameters, i.e., a parameter-free optimization algorithm. Even though the preceding experimental findings demonstrate that the RCOA outperforms the majority of comparison algorithms, it even has significant downsides that must be considered. The effectiveness of the RCOA can be enhanced, but there is always an opportunity for improvement. It is also discovered that the RCOA can achieve theoretically optimal values in the unimodal benchmark function despite the minimal number of theoretically optimal results calculated by the RCOA, indicating that its exploitation ability in the unimodal test function could be further enhanced. Upon additional testing of the multimodal benchmark function, it is discovered that the number of interesting optimum values achieved is limited, indicating that the RCOA's exploration and exploitation capabilities under multiple optimal solutions require more improvement. The RCOA, on the other hand, has not been confirmed in a higher-dimensional context. To summarize, the advantages and limitations of the RCOA are given below.

The merit of the approach can be concluded based on a few key points:

- The RCOA performed competitively when benchmarked against 23 benchmark test problems and eight real-world design optimization problems. It demonstrated superior convergence rates and solution quality in several instances, underlining its efficacy and robustness.
- The RCOA is a physics-inspired algorithm that leverages the fundamental principles of a resistance–capacitance series circuit. The novelty of the algorithm lies in its simplicity and the innovative way it mimics both the transient and steady-state response of the circuit.
- The approach is not problem-specific and can be applied broadly across various industrial engineering computation problems.

As with any approach, the RCOA also has certain limitations:

- While the RCOA performed well across the benchmark and real-world problems, its effectiveness may be reduced with more complex, higher-dimensional optimization problems. Future work is needed to explore and improve its performance in such scenarios.
- Like many optimization algorithms, our approach requires careful parameter tuning, i.e., selection of population size and maximum number of iterations, to ensure optimal performance. We have provided some guidelines in the paper, but tuning may need to be adapted based on the specifics of the problem at hand.
- For large-scale problems, the computational cost may be a constraint due to the iterative nature of the algorithm. More research is required to reduce computational overhead and make the RCOA more efficient for larger problem instances.

## Conclusions and future scopes

This study proposed the resistance–capacitance optimization algorithm, a revolutionary optimization algorithm, and its performance is deeply investigated using various numerical and practical engineering optimization problems. The properties of the proposed RCOA are as follows.

- RCOA is inspired by the principles of physics, specifically the behaviour of a Resistance–Capacitance series circuit. The algorithm effectively mimics both the transient and steady-state responses of such a circuit.
- RCOA is a population-based method that optimizes stochastic search processes, making it flexible and adaptable to various problems.
- The algorithm employs mechanisms to balance exploration and exploitation. This property is vital for maintaining diversity in the population and avoiding premature convergence.
- RCOA has proven to be a reliable algorithm, as demonstrated in our study by its competitive performance on both benchmark and real-world problems. RCOA can escape local optima and converge to the global optimum.
- The algorithm has been designed to be scalable and applies to problems of different dimensions. However, as with any algorithm, there may be trade-offs with computational efficiency as the problem size increases.
- As with many optimization algorithms, RCOA involves parameter tuning, i.e., population size and maximum number of iterations, to achieve optimal performance. However, the algorithm was designed to minimize the sensitivity to these parameters to the greatest extent possible.
- RCOA’s structure and operations are relatively simple compared to other optimization algorithms. This simplicity, in turn, contributes to its computational efficiency.

The suggested algorithm was evaluated using the 23 traditional benchmark test functions, as well as eight real-world constrained engineering design optimization problems, to investigate its effectiveness. A penalty function-based constraint handling mechanism was employed in the proposed RCOA to solve real-world constrained engineering design problems. To make an even more meaningful comparison, the proposed RCOA is compared to other algorithms at all phases of experimentation. Aside from that, the Friedman’s ranking test was used to rank the algorithms. Among all the chosen algorithms, the RCOA came out on top for numerical and constraint engineering design optimization problems based on the simulation and statistical non-parametric assessment results. The boxplot analysis and convergence curves demonstrated the robustness, reliability, accuracy, efficiency, and applicability of the RCOA for numerical and engineering design optimization problems. Based on RT values and convergence graphs, the convergence and computational time are significantly superior to the chosen algorithms. In conclusion, the suggested RCOA is a trustworthy and simple optimization method for numerical and engineering design optimization problems.

There is much scope for future extensions and applications for any new research. Based on this statement, there is much scope for the proposed RCOA in different fields of applications, such as economic load dispatch, economic emission dispatch, optimal power flow, FACTS device placements, unit commitment, harmonic distortion minimization, wind farm layout design, BLDC motor design, load forecasting, solar and wind power forecasting, feature selection, image enhancement, image segmentation, machine learning, artificial intelligence, wireless sensor networks, etc. When any new algorithm is applied to different real-world applications, there may be a chance of getting trapped at local optima. In order to avoid the local optima trap, the proposed RCOA may also be improved by techniques such as trap-avoiding operators, Levy flight mechanism, Chaos theory, orthogonal learning, opposition-based learning, etc. In addition, the many- and multi-objective versions of the proposed algorithm are another scope for improving the RCOA.

## Data Availability

The datasets used and/or analysed during the current study are available from the corresponding authors upon reasonable request. The basic MATLAB code of the algorithm will be shared from the corresponding authors upon reasonable request.

## List of symbols

${I}_{1}$, ${I}_{2}$, ${I}_{3}$, ${I}_{4}$, ${I}_{5}$, and ${I}_{6}$: Branch currents
$v\left({0}^{-}\right)$: Capacitor voltage before closing the switch
$v\left({0}^{+}\right)$: Capacitor voltage after closing the switch
${V}_{s}$ and $v$: Source voltage and initial voltage, respectively
$R$ and $C$: Resistance and Capacitance, respectively
$\frac{dv}{dt}$: Rate of change of voltage\
${v}_{0}$: Voltage at time *t* = 0
$\tau$: Time constant of the RC circuit
${v}_{natural}$ and ${v}_{forced}$: Natural and forced voltage responses
${v}_{ts}$ and ${v}_{ss}$: Transient and state-state responses
$v\left(\infty \right)$: Final capacitor voltage
$$it$$: Current iteration
$Maxit$: Maximum number of iteration
$$a$$: Constant (= 5)
$b$ and ${r}_{1}$: Uniform random number between [0,1]
$\overrightarrow{{X}_{ts}}$ and $\overrightarrow{{X}_{ss}}$: Position vectors (voltage) of the transient-state and steady-state, respectively
$$X$$: Population position
$ub$ and $lb$: Upper bound and lower bound of the decision vectors
$rand$: Random number between $[\mathrm{0,1}]$
${f}_{obj(best)}$: Best objective function values
$$dim$$: Problem dimension
$$N$$: Population size
